# Tissue tropism, pathology, and pathogenesis of West Nile virus infection in saltwater crocodile (*Crocodylus porosus*)

**DOI:** 10.1371/journal.pntd.0013385

**Published:** 2025-08-04

**Authors:** Gervais Habarugira, Nicholas K. Y. Yuen, Willy W. Suen, Jasmin Moran, Jody Hobson-Peters, Roy A. Hall, Sally R. Isberg, Helle Bielefeldt-Ohmann

**Affiliations:** 1 School of Veterinary Science, The University of Queensland, Gatton, Qld, Australia; 2 School of Chemistry and Molecular Biosciences, The University of Queensland, St Lucia, Qld, Australia; 3 Centre for Crocodile Research, Noonamah, Northern Territory, Australia; 4 Australian Infectious Diseases Research Centre, The University of Queensland, St Lucia, Qld, Australia; Pontificia Universidad Catolica de Chile, CHILE

## Abstract

West Nile virus (WNV) is one of the leading causes of economic losses to the saltwater crocodile farming industry due to skin lesions, known as “pix”, induced by the infection. Our previous study suggested a possible immunopathological pathway causing these lesions. We therefore resolved to investigate the kinetics of WNV-infection and the elicited immune responses in experimentally challenged saltwater crocodile hatchlings. Employing virus isolation, quantitation of viral genome loads in tissues by RT-qPCR and immunohistochemistry, we demonstrated that upon infection, the virus replicates in the spleen, liver and later in the pancreas. Transcriptomic analysis, based on RNA sequencing and RT-qPCR of kidney and liver tissues, revealed that the early host response is primarily via alteration of cellular structure and metabolism. As the infection progresses, the response becomes predominantly inflammatory and antiviral. The results suggest that the kidney and gastrointestinal tract are primary nidi of viral replication leading to cloacal shedding, but a link to skin lesion development remains to be fully clarified.

## Author summary

West Nile virus (WNV) infection in crocodilians has been associated with disease and skin lesions known as “pix.” These lesions result in significant economic losses for the crocodile farming industry in Australia, the USA, and Africa. In this study, we aimed to understand the mechanisms that may lead to lesion development following WNV infection. Through experimental infection, we confirmed the presence of the virus and its replication in various body systems, including the digestive and urinary systems. Our findings suggest that the virus primarily replicates in the kidneys and digestive system, which leads to shedding through the cloaca. However, the mechanism behind the development of skin lesions still requires further investigation.

## Introduction

West Nile virus (WNV), an orthoflavivirus and zoonotic arbovirus, is now almost globally ubiquitous. WNV infections and diseases have been reported in several animal species, including humans, horses, sheep, birds, and reptiles [1–4]. Infected hosts display a broad spectrum of clinical presentations ranging from subclinical to severe neurological forms [4]. Susceptible hosts such as humans, horses and American alligators may experience severe neurological, digestive, renal, and cutaneous manifestations [4–7]. In contrast, saltwater crocodiles (*Crocodylus porosus*) only present with skin lesions following WNV infection [8]. Under farming conditions, 10 – 30% of saltwater crocodiles may develop skin lesions, leading to skin depreciation and rejection by the tanning industry [9].

Although lesions caused by infectious agents other than WNV, may also resemble pix, such as those caused by Crocodyline herpes virus, they tend to occur in conjunction with other clinical signs such as conjunctivitis and pharyngitis as well as systemic lymphoid proliferation with nonsuppurative encephalitis [10,11]. The WNV-induced pix lesion consists of lymphoid aggregates in the dermis, causing irreversible collagen degeneration with or without thinning of the overlaying epidermis, but no breach of the Stratum corneum. Interestingly, while similar lymphoid aggregates were also observed in the gastrointestinal tract of WNV experimentally infected hatchling crocodiles, no viral antigen was detected in any of the lesions by immunohistochemistry [8]. Despite this, viral RNA was detected in cloacal swabs and water samples, suggesting virus shedding into the water [8]. In reptilians, the waste products of the gastrointestinal and urinary systems enter a single chamber called the cloaca and are excreted via the vent [12,13]. Interestingly too, even though the experimentally infected animals were never exposed to natural infection via mosquito bites or experimentally re-infected, neutralizing antibody titers kept rising over several months post infection suggesting possible persistence of virus in some tissues or continuing immune maturation. However, very little is known about the crocodilian innate and adaptive immune response to viral infections [14].

The diagnosis and control of disease in any given host requires a thorough understanding of the pathogenesis, including pathogen tissue tropism, infection kinetics and innate immune responses, i.e., host-pathogen interaction [15–17]. Our previous study did not unambiguously resolve the tissue tropism of virus replication in infected saltwater crocodiles [8]. However, based on our findings we hypothesized that the skin lesions are of immunopathological character, resulting from a strong innate immune response during acute viral infection followed by a virus-clearing adaptive immune response [8,18]. In this study we aimed to elucidate the WNV infection kinetics and tissue tropism in the saltwater crocodiles and to further characterize the role of innate and adaptive immunity in WNV infection.

## Materials and methods

### Ethics statement

The study was conducted in accordance with the Charles Darwin University research ethics guidelines. Ethical approval for the experiment was obtained from Charles Darwin University Animal Ethics Committee (A19027, A18004). The Centre for Crocodile Research has a license with the animal welfare unit of the Northern Territory Government to conduct research on crocodilians (permit no. 061). During infection and sample collection, animal care and use protocols adhered to the Animal Welfare Regulations 2000 of the Northern Territory of Australia and Code of Practice on the Humane Treatment of Wild and Farmed Australian Crocodiles [19].

### Animals and experimental design

A total of 98 hatchlings were used in this experiment. They were obtained from different wild egg clutches hatched under standard incubation conditions at a commercial crocodile farm. Animals were given individual identification marks by scute cutting and tagging as previously described [8]. Crocodile hatchlings were tested regularly for anti-WNV maternal antibodies until they were free of WNV-neutralizing antibodies, generally when they were about four months old. The hatchlings were then transferred to a mosquito-free biosecurity level 2 (BSL2) facility at Berrimah Veterinary Laboratory (BVL) in Berrimah, the Northern Territory, Australia.

**Fig 1:**
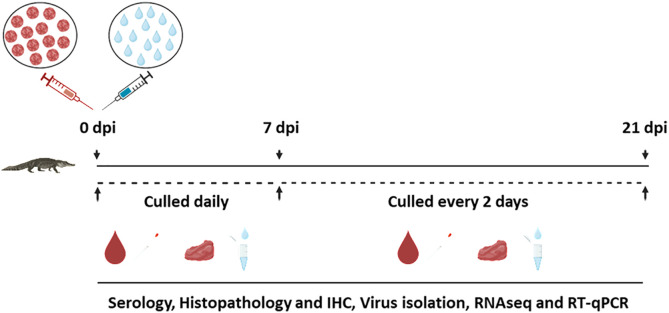
An illustrative summary of WNV infection kinetics experiment in saltwater crocodile hatchlings. Samples, including blood, tissues, cloacal swabs (collected at necropsy) and water were collected daily for the first 7 dpi and every second day from day 9 dpi. Created with BioRender.com.

Crocodile hatchlings free of anti-WNV maternal antibodies were infected by subcutaneous injection at the tail base with 1 x 10^5^ IU of a Kunjin strain of West Nile virus (WNV_KUN_), cultured and suspended in 100 µL of sterile PBS as previously described [8]. The control group was sham infected with 100µL of sterile PBS (mock-infected) and housed in a separate pen within the same mosquito-free facility. A number of uninfected animals were also placed in pens with the infected animals (in–contact controls) (S1 Table and Fig 1).

All animals were monitored daily for clinical signs or changes in behavior. For the first 7 days post infection (dpi), a predetermined, randomly selected set of infected animals (n = 5), mock-infected control animals (n = 0 or 2) and or in–contact control animals (n = 0 or 4) were euthanized, and various samples collected for testing (Fig 1). After 7 dpi, the remaining animals were euthanized and sampled every two days (n = 5 for infected animals; variable numbers for mock-infected and in-contact control animals) until the end of experiment at 21 dpi (S1 Table and Fig 1).

### Blood, tissue, cloacal swabs, and water sampling

Blood was collected from all hatchlings immediately prior to euthanasia, followed by necropsy and tissue collection. Blood samples were aseptically collected from the occipital sinus using a 23-gauge needle and vacutainer tubes without anticoagulant (Becton Dickinson, Australia). The procured serum samples were tested in blocking ELISA and virus neutralization test as described previously [20]. Cloacal, water and oral swab samples were collected and processed and tested by RT-qPCR as previously described [8].

Tissue samples were collected to measure viral load in tissues by infectious virus (traditional TCID_50_ titration) and indirectly by quantifying WNV nucleic acid by RT-qPCR, transcriptomic analysis by RNA sequencing, and assessment of pathology by microscopy and immunohistochemistry. Samples collected for infectious virus titration, transcriptomics analysis and cytokine transcriptional profiling included liver, spleen, kidney, oral cavity mucosa, the glosso-epiglottic fold mucosa, skin and the third eyelid. Samples for histopathology examination included brain, eye, tongue, lung, heart, gastrointestinal tract (esophagus, stomach, small and large intestines, and the cloaca), liver, pancreas, spleen, kidney, and skin.

For infectious virus isolation, about 0.5 cm^3^ of sample was collected aseptically at post-mortem from the above listed tissues and snap-frozen on dry ice. The samples were then stored at -80^o^C until tested. A tissue sample of a similar size was collected, stored in RNAlater solution (CAT AM7021, Invitrogen Thermo Scientific, USA), and kept at –20^o^C until processed for RNA extraction.

### Histopathology and immunohistochemistry (IHC)

Crocodile tissue samples were processed and examined as previously described [8]. Briefly, collected tissue samples were fixed in 10% neutral buffered formalin for 48 hours before being transferred into 70% ethanol and stored until they were trimmed and routine paraffin embedded. Sections (4–5 µm) were either stained with hematoxylin and eosin or subjected to immunohistochemical detection of viral antigen using monoclonal antibody 4G4, specific for flavivirus NS1 [21]. Digital microphotographs were taken using a Nikon DS-Fi1 camera with a DS-U2 unit and NIS elements F 4.60 software and are presented without manipulation other than adjustment for brightness.

### RNA extraction

#### a) RNA extraction from tissues.

RNA was extracted from tissues using Qiagen RNeasy Plus Mini Kit (CAT 74136 Qiagen, Hilden, Germany) according to the manufacturer’s instructions. Briefly, about 30 mg of tissue was homogenized in a 2 mL microcentrifuge tube containing a 5 mm diameter stainless steel bead (CAT 69989, Qiagen, Hilden, Germany) and 0.6 mL of RLT lysis buffer. The tissue was lysed using Qiagen TissueLyser II (Qiagen, Hilden, Germany) at 30 Hz for 2 min. Then the tubes were centrifuged at 12,000 x *g* for 5 min at room temperature. The supernatants were transferred to gDNA Eliminator spin columns placed in a 2 mL collection tube. The subsequent steps were performed according to the Qiagen RNeasy Plus Mini Kit protocol. Prior to RNA sequencing, genomic DNA digestion was performed on isolated RNA using DNase I digest (CAT 79254, Qiagen, Hilden, Germany) in solution with RNA clean up using RNeasy Mini Kit (CAT 74106, Qiagen, Hilden, Germany).

#### b) RNA extraction from blood plasma and cloacal swabs.

Viral RNA was extracted from plasma and swab samples using the Machery-Nagel Viral RNA Isolation kit (CAT 740956.250, Dueren, Germany) as per the manufacturer’s instructions. The quality and quantity of purified RNA were assessed by Nanodrop (Thermo Scientific NanoDrop One Spectrophotometer).

### Virus quantitation in blood and tissues

Virus isolation and quantitation in blood and tissues were done directly by TCID_50_ assay and indirectly by RT-qPCR as previously described [8]. The viral titers in blood and tissues were expressed as TCID_50_ and TCID_50_ equivalent/mL and TCID_50_ or TCID_50_ equivalent per gram of tissue sample, respectively. The inoculum was added onto Vero cells (African green monkey (*Chlorocebus aethiops*) kidney epithelial cells) and BSR cells (Golden hamster (*Mesocricetus auratus*) baby kidney epithelial cells) monolayer and incubated for two hours after which the inoculum was removed, and the monolayer washed with sterile PBS. Fresh DMEM (CAT 11960069, Gibco, Thermofisher Scientific) media supplemented with 5% fetal bovine serum (FBS), and penicillin-streptomycin-glutamine mix was subsequently added, and the culture plates were incubated as previously described [18,20].

Due to high levels of *in vitro* cell death caused by crocodile plasma toxicity, even when the inoculum was removed two hours post-infection, we were unable to quantify viremia by the standard TCID_50_ titration on cells and only TCID_50_ equivalent/mL are presented for blood.

### Antibody responses

The post-infection adaptive immunity was assessed by virus neutralization test (VNT) as previously detailed [20]. The antibody titers were expressed as the reciprocal of the highest serum dilution where the virus replication was completely neutralized.

#### a) RNA sequencing library preparation and sequencing.

The analysis involved the transcriptomic differential analysis in early and late stages of infection. Early timepoints included samples collected between day 1 and day 6 post infection, which were further subdivided into timepoints 1 (1 – 2 dpi) and 2 (3 – 6 dpi). Late timepoints included samples collected between day 7 and day 21 post infection, which were subdivided into two time points, with the first time point being 7 – 9 dpi and the second running between 11 – 21 dpi. RNA sequencing and analysis was performed in R Studio and the Galaxy Australia platform as described previously [22] using the NCBI publicly available *Crocodylus porosus* reference genome (GCF_001723895.1, BioProject: PRJNA163131, version 13 Sept 2016) [23]).

#### b) Cytokine gene expression profiling assay by RT-qPCR.

RNA extracted from tissues was reverse transcribed into cDNA using qScript cDNA Synthesis Kit in 20 µL (CAT 95047–100, Quantabio Reagent Technologies, Massachusetts, USA USA). The cDNA synthesis consisted of a 20 µL reaction containing 4 µL of 5x qScript reaction mix (containing a mix of oligo-dT and random primers), 1 µL 20x qScript reverse transcriptase, 4 µL of RNA template, and 1 µL RNAseOUT (ThermoFisher, USA) and 10 µL of nuclease-free water. The reverse transcription consisted of one cycle at 22°C for 5 minutes, one cycle at 42°C for 30 minutes, and one cycle at 85°C for 5 minutes. cDNA was diluted two-fold in nuclease free water before it was tested by qPCR.

qPCR was performed on cDNA using QuantiNova SYBR Green PCR Kit (CAT 208052, Qiagen, Hilden, Germany) in 20 μL reactions. The 20 μL reaction was made of 10 µL of 2x QuantiNova SYBR Green PCR Master Mix, 2 µL of cDNA template, and 0.7 µM of each primer. The remaining volume was made up of nuclease-free water. Tissue samples from three animals (biological replicates) were used for the first seven timepoints after which only one animal was used per timepoint. For each qPCR run, positive controls from *in vitro* infected crocodile liver and kidney-derived cells (1-LV and 4CPK) were used [8,24]. No template control and two negative control samples collected from uninfected hatchlings were used in each run.

The qPCR cycling conditions comprised one cycle at 95°C for 2 minutes followed by 40 cycles at 95°C for 5 seconds and 60°C for 10 seconds. The melting curve analysis was performed between 72°C and 95°C. PCR specificity was determined by assessing the number and magnitude of peaks in the melt curve as compared to those in the no template and negative controls. PCR products were analyzed on 2% agarose gel to confirm the expected amplicon size. The target cytokine genes included antiviral effector genes and ISGs (*irf3, oasl, and mx1*), pro- and anti-inflammatory genes (**il1*β, *il34, hif-1*α, *csf-1[m-csf],** and **tgf*β*), as well as cell proliferation and apoptosis (*ki67* and *casp 9*) genes.

The fold changes in cytokine genes expression were calculated using the 2^-∆∆CT^ formula as previously described [25]. Briefly, this involved calculating the ΔCT value by subtracting the CT value of the housekeeping gene from the CT value of the target gene. Delta delta CT (ΔΔCT) was calculated by subtracting the average of control ∆CT from the ∆CT of the infected sample. Eight control samples across four timepoints (1, 7, 13 and 19 dpi) were used for the averaging of the control ∆CT. Pairwise comparisons between samples and timepoints was performed using two-way ANOVA with post-test Tukey’s multiple comparison. P < 0.05 was considered statistically significant.

## Results

### Virus titration from serum

**Fig 2:**
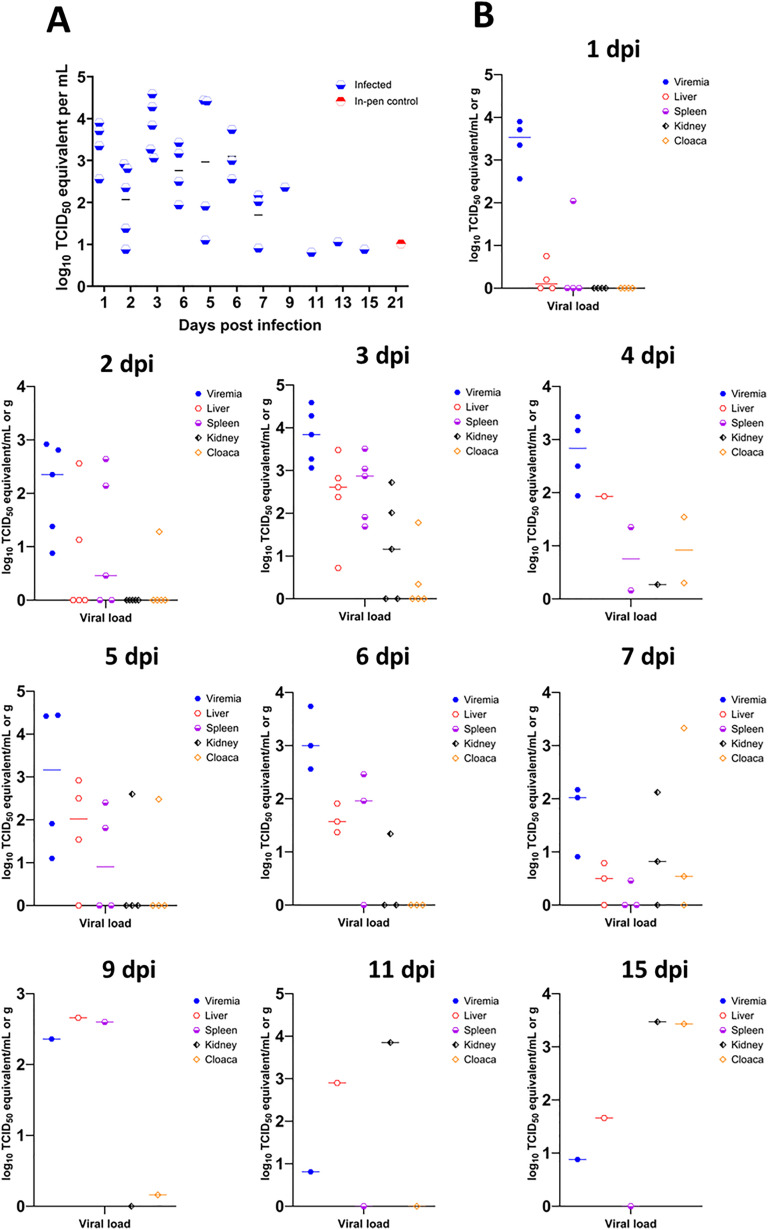
Viremia and viral load in selected tissues of infected saltwater crocodiles in relation to viremia measured by RT-qPCR at various timepoints. A. Viremia per timepoints expressed in log10 TCID_50_ equivalent per mL of blood. The number of animals euthanized per time point is summarized in S1 Table. B. Viral load in liver, spleen, kidney and cloacal mucosa in relation to viremia in each timepoint. Only tissues from animals with viremia were examined. The horizontal bars indicate median values.

**Fig 3:**
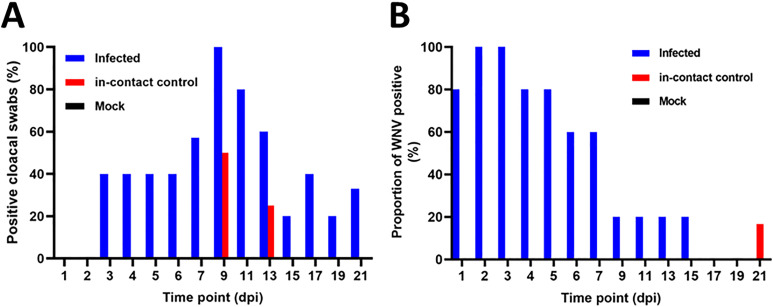
Proportion of WNV positive among infected, in-contact control and mock-infected crocodiles. A. Proportion of cloacal swabs positive for WNV by RT-qPCR at different time points post experimental WNV challenge. B. The proportion (%) of crocodile hatchlings positive for WNV by RT-qPCR in blood at various time points post-infection.

Viral RNA was detected in most serum samples collected from infected animals and one of the in–contact controls from one dpi until end of experiment at 21 dpi (Fig 2 A and 2B). The highest individual viral titer was 10^4.59^ TCID_50_ equivalent/mL while the highest average viral titer was 10^3.81^ TCID_50_ equivalent/mL, observed in tissues collected from infected crocodiles terminated at 3 dpi (S3 Table). At least 60% of the infected animals were viremic in the first 7 – 9 dpi (Fig 2A). At 1 dpi, the viraemia titer was higher than on day two (S2 Table). By 21 dpi, 20% of in- contact animals had become viremic (Fig 3). None of the mock-infected animals became viremic.

### Virus detection and quantitation by qRT-PCR in tissues

Viral RNA quantitation was performed on the liver, spleen, kidney, cloacal mucosa, the glosso-epiglottic fold mucosa, skin and the third eyelid samples. Only tissue samples from viremic animals were tested. Viral RNA was detected as early as 1 dpi in the liver and spleen, 2 dpi in kidney, 3 dpi in cloacal mucosa (Fig 2B). Only one eyelid sample collected on 5 dpi was positive for viral RNA by RT-qPCR at 10^2.7^ TCID_50_ equivalent/g. Neither the glosso-epiglottic fold mucosa nor skin were positive for RT-qPCR at any timepoint.

Samples positive for WNV viral RNA were subjected to virus isolation by inoculation onto Vero and BSR cells. Some of those samples yielded infectious virus (S2 Table); however, due to toxicity of the samples for both mammalian cell lines the limit of detection was high (10^3.80^ TCID_50_/g), likely resulting in many false negative samples.

### Virus detection by RT-qPCR in cloacal swabs

Cloacal swab samples were collected from each saltwater crocodile at termination. WNV viral RNA was first detected 3 dpi in virus challenged animals. Viral RNA was detected in 40% of the infected animals euthanized between 3 and 6 dpi. All infected animals euthanized at 9 dpi were positive for WNV RNA in the swabs. The first in–contact control RNA positive cloacal swabs were detected at 9 dpi, where half of the in–contact control animals were positive for viral RNA (Fig 3A). From 11 dpi, the proportion of RNA positive cloacal swabs in both infected and in–contact controls decreased until the end of the experimental period at 21 dpi (Fig 3A).

### Pathology and immunohistochemistry

**Fig 4:**
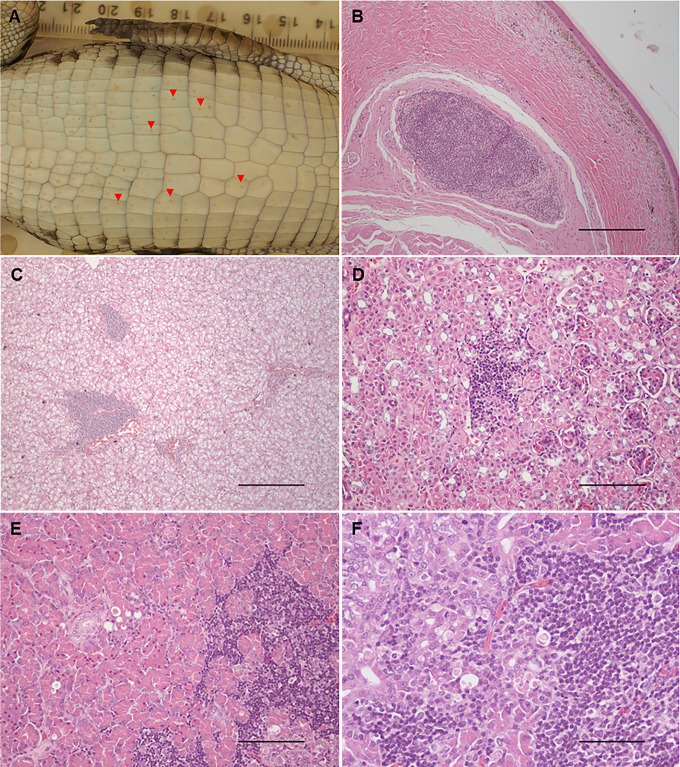
Lymphoid aggregates in different tissue sections. A. Macroscopic “pix” lesions (red arrow heads) in the skin of an infected crocodile. B. Lymphoid aggregates in a skin from an infected crocodile. C. Periportal lymphoid aggregates in the liver with mononuclear infiltration in periportal zones. D. Interstitial lymphoid aggregates the in kidney. E and F. Exocrine pancreatic cell degeneration with interstitial mononuclear infiltration. Scale bars: A and C 625 µm; D and E 125 µm; F 65 µm.

Gross skin lesions were noted in four animals at necropsy on 5, 6 and 17 dpi, respectively (Fig 4). However, only one animal from the experimentally challenged group, at 17 dpi, had a typical pix lesion, which was confirmed histologically (Fig 4A and 4B). Histologically, focal to multifocal lymphoplasmacytic and histiocytic infiltrates as well as lymphoid aggregates were observed in multiple tissues including conjunctiva, liver, spleen, kidney, stomach, small and large intestines, cloacal mucosa, and pancreas at most time points (Fig 4C–F).

**Fig 5:**
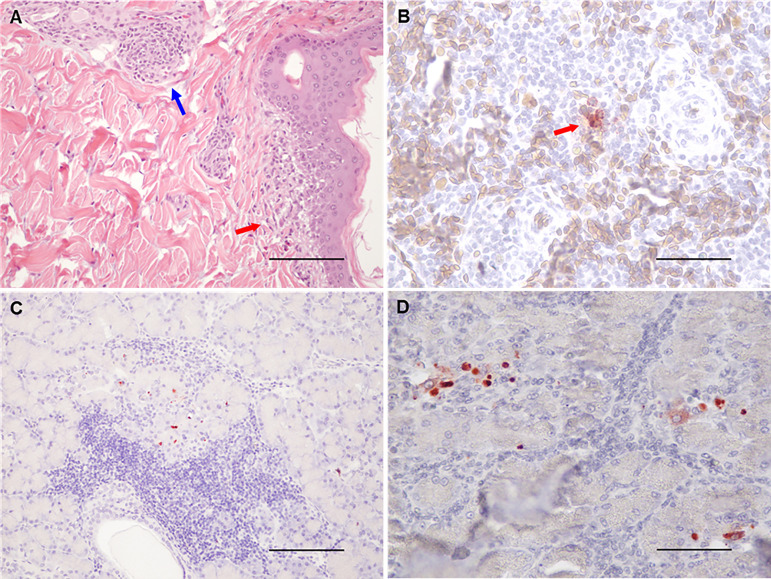
Histopathological lesions in infected saltwater crocodiles. A. Mild intraepithelial and epidermal infiltration of mononuclear cells (lymphocytes and monocytes) (red arrow), mild perivascular lymphocyte infiltration in the dermis (blue arrow), scale bar 1250 µm. B. Immunohistochemical detection of WNV NS1, using mAb 4G4, in spleen from a crocodile infected with WNV_KUN_. There are viral protein-positive monocytes (red arrow), scale bar 125 µm. Note that the brown round oval cells in panel B are nucleated erythrocytes. C. and D. Immunohistochemical detection of WNV NS1 (red signals) in exocrine pancreas from animals terminated 13 and 15 dpi, scale bars 250 and 65 µm, respectively.

Two animals terminated at 3 dpi, one animal terminated at 5 dpi, two animals terminated at 6 dpi and one terminated at 7 dpi had mild intraepithelial and subepidermal infiltration of lymphocytes and monocytes, and mild perivascular lymphocyte infiltration in the dermis (Figs 4B and 5A). However, none of these skin samples tested positive for viral nucleic acid, and no viral antigen was detected by immunohistochemistry (IHC).

Viral antigen was detected by IHC using anti-flavivirus NS1 monoclonal antibody 4G4 in the spleen of two animals terminated on 3 and 5 dpi, respectively, and in three pancreas samples from animals terminated at 13 dpi (n = 1) and 15 dpi (n = 2) (Fig 5C and 5D). The latter three animals had detectable WNV-neutralizing antibodies at the time of termination.

### Antibody responses

**Fig 6:**
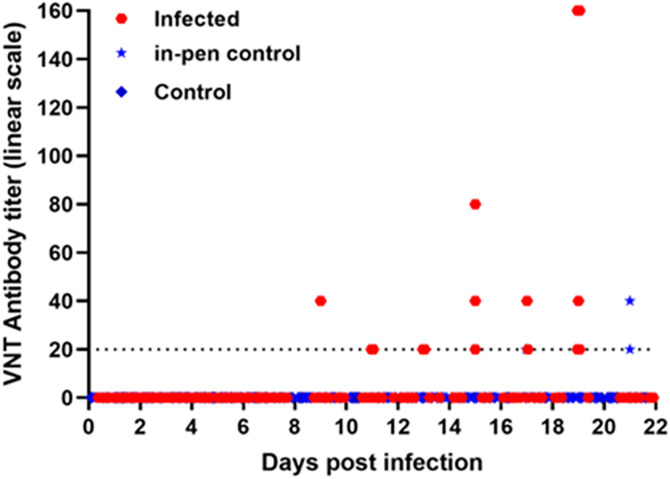
Neutralizing antibody titers in individual experimentally infected and in-pen contact hatchling saltwater crocodiles. The dotted line represents the limit of detection at a titer of 1:20 dilution.

Virus neutralizing antibodies were first detected in the virus-challenged saltwater crocodiles at 9 dpi with titers in the range of 20–160 (Fig 6). Three in-pen contact animals had developed neutralizing antibodies by 17 dpi, with antibody titers ranging between 20 and 40 (Fig 6). None of the mock-infected animals had detectable WNV-neutralizing antibodies.

### Transcriptome associated with WNV infection

**Fig 7:**
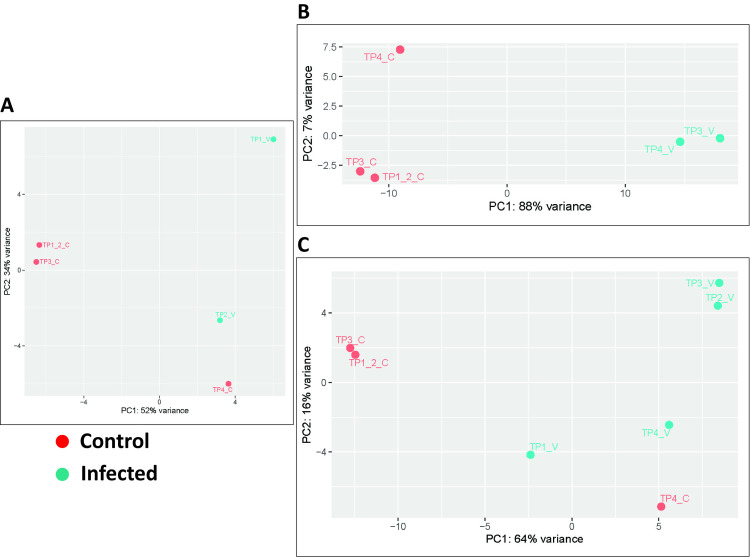
Principal component analysis (PCA) plots for RNA-seq data of the kidney and liver. A, B. PCA plots for RNA-seq data of the kidney at various time points during early and late response to infection. C. PCA plots for RNA-seq data of the liver at different timepoints of late response to infection.

Based on the above results, we examined the transcriptome of the crocodile liver, kidney, and spleen during early (1 – 6 dpi) and late (7 – 21 dpi) phases of infection using next-generation RNA sequencing and differential gene expression analysis. Liver and kidney samples yielded quality sequences, but all spleen samples failed the post-sequence quality control check and were excluded from further analysis. Samples from WNV–infected and mock infected animals yielded distinct transcriptomic expression as demonstrated by principal component analysis where the control and infected samples clustered separately for both liver and kidney (Fig 7). The interpretation of transcriptomics kinetics relative to infection progression was based on gene ontology, heatmap, and principal components analyses. In cases where over 80 genes were differentially expressed, the heatmap analysis focused exclusively on the top 80 differentially expressed genes (either upregulated or downregulated) in each tissue.

### Transcriptome associated with early and late phase of WNV infection in kidney

**Fig 8:**
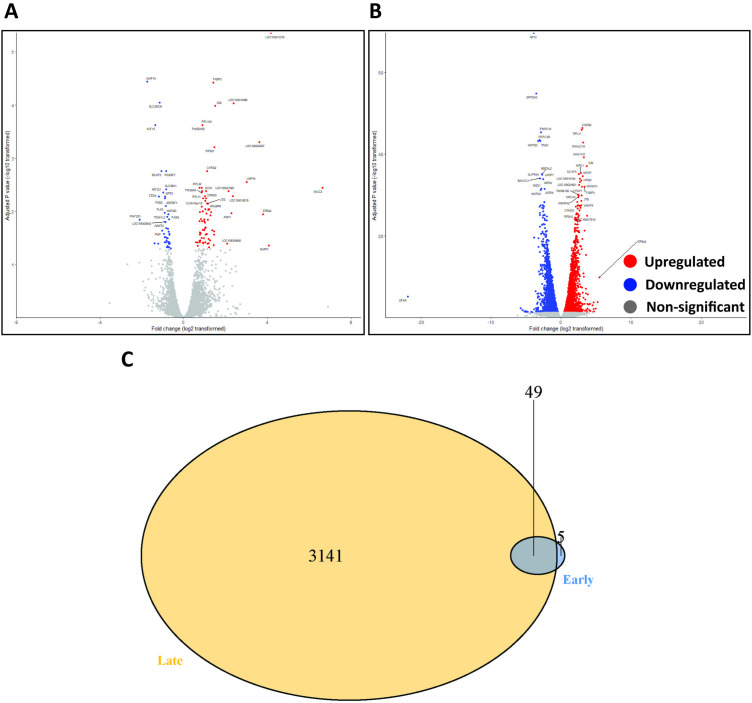
Distinct transcriptional signatures in the kidney during early and late response to infection. (A and B) Volcano plot for early and late response to infection in kidney. Blue dots indicate downregulated genes, red dots indicate upregulated genes, grey dots indicate non-significantly expressed genes. (C) Venn diagram of genes differentially expressed during early (blue area, n = 54) and late (yellow area, n = 3141) response to infection. The dark blue overlap corresponds to the genes (n = 49) that were expressed during both phases of infection.

**Fig 9:**
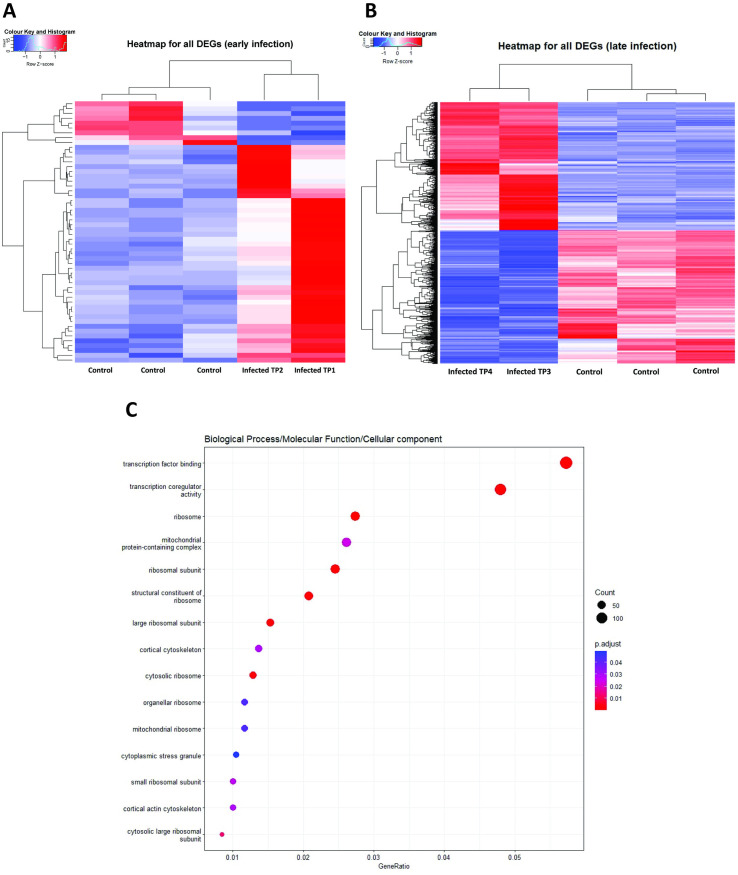
Enriched gene ontology associated with transcriptional response in kidney during early and late response to infection. (A and B) Heatmaps of all significant differentially expressed genes in the kidney (early and late response to infection). (C) Gene Ontology (GO) enrichment analysis of genes differentially expressed in the kidney during the late response to infection. TP means timepoint.

Compared to mock-infected controls, 118 and 5,195 genes differentially expressed during the early and late response to infection, respectively (Figs 8A, 8B, S1A and S1B). Of the 118 differentially expressed genes during early response to infection, 75 genes were upregulated while 43 were downregulated (Fig 9A). During the late response to infection, 2,552 genes were upregulated while 2,643 were downregulated.

Out of the significantly differentially expressed genes, five genes were significantly differentially expressed (*p* < 0.05, log 2-fold change > 1) only during the early response to infection, while a total of 3,141 genes were significantly differentially expressed only in the late response to infection. Forty-nine genes were significantly differentially expressed during both early and late responses to infection (Fig 8C).

Gene ontology (GO) enrichment analysis did not yield any results for early response to infection. The heatmap analysis of the significantly differentially expressed genes revealed genes involved in regulating mitochondrial (e.g., MRPS24, DAP3, DNAJC15, DNAJC19) and ribosomal structure and metabolism (e.g., RPL11, RPL10A, RPS15, MRPS31, MRPS14, MRPS18C) were upregulated. Genes that regulate organellar small ribosomal subunit (e.g., RPS15, RPS27L, RPS3A, RPS14) were downregulated (Fig 9A). Genes relating to inflammation, repair, apoptosis and antiviral responses (e.g., IFI27L2, IFIT5, CXCL10-like,) were upregulated in the per-acute phase of infection (1–3 dpi) and continually increased in its transcription to 4–6 dpi. Interestingly, transcription of genes relating to mitochondrial functions (e.g., IFI27L2a, RPS21, CARS2) were markedly upregulated only during the per-acute phase of infection (1–3 dpi), but not in 4–6 dpi (Fig 9A).

In the late response to infection (7–21 dpi), GO enrichment analysis showed that a number of genes associated with the regulation of transcription factor binding (e.g., SPEN, CTDP1, NSD1, NCOA1), transcription coregulator activity (e.g., SPEN, NSD1, PFDN5, NCOR2), and mitochondrial and ribosomal metabolism (e.g., MRPS24, DAP3, MRPS6, MRPL48) were upregulated. In contrast, downregulated genes are involved in organellar ribosome (e.g., MRPS24, DAP3, MRPS6, MRPL48), mitochondrial ribosome (e.g., MRPL32, MRPL41, MRPS28, MRPL43), and cytoplasmic stress granules (e.g., LARP1, EIF4G1, NUFIP2, HIPK2) (Fig 9C and S4 Table). Interestingly, based on the heatmap, it appears that transcription of genes relating to cell proliferation, apoptosis and cellular homeostasis (e.g., MAGOH, NFYB, CDC14A, CD3D, DAP) were markedly upregulated in the third stage of infection (7–9 dpi), but the level of upregulation was reduced in the fourth stage of infection (11–21 dpi). In line with our understanding of a more slowly developing adaptive immune response in reptiles, transcriptions of genes related to T-cell responses to viral infection and mediation of IFN-γ-induced cell death (e.g., DAP, DCAF5) continue to increase between the third (7–9 dpi) and fourth stage of infection (11–21 dpi) (Fig 9B).

### Transcriptional profiles associated with early and late responses to WNV infection in liver

**Fig 10:**
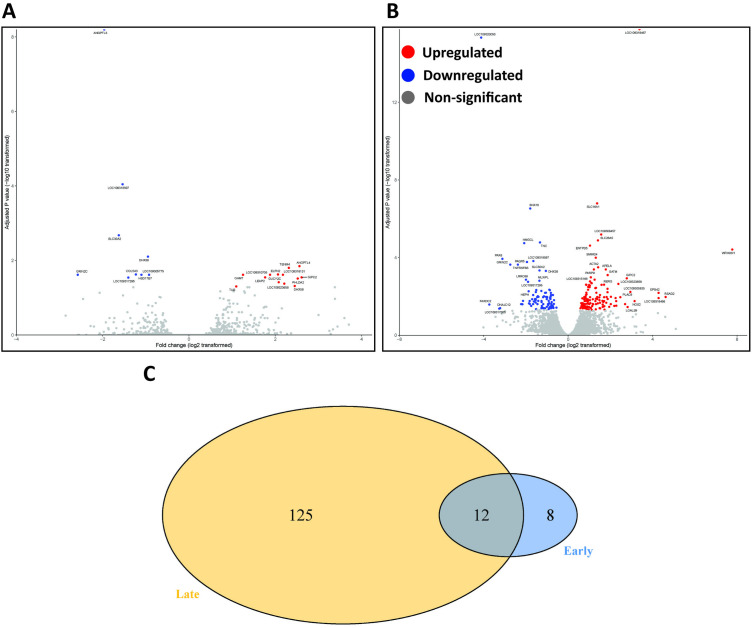
Distinct transcriptional signatures in liver during early and late response to infection. (A and B) Volcano plot for early and late response to infection in liver. Blue dots indicate downregulated genes, red dots indicate upregulated genes, grey dots indicate non-significantly expressed genes. (C) Venn diagram of genes differentially expressed during early (blue area, n = 20) and late (yellow area, n = 125) response to infection. The dark blue overlap corresponds to the genes that are expressed during both phases of infection (n = 12).

The analysis of transcriptomes in liver tissues identified eight genes that were significantly differentially expressed solely during the early response to infection, and 125 genes that showed significant differential expression changes only during the late response. Additionally, there were 12 genes that were differentially expressed in both the early and late stages of the infection response, indicating their involvement in both phases (Fig 10).

**Fig 11:**
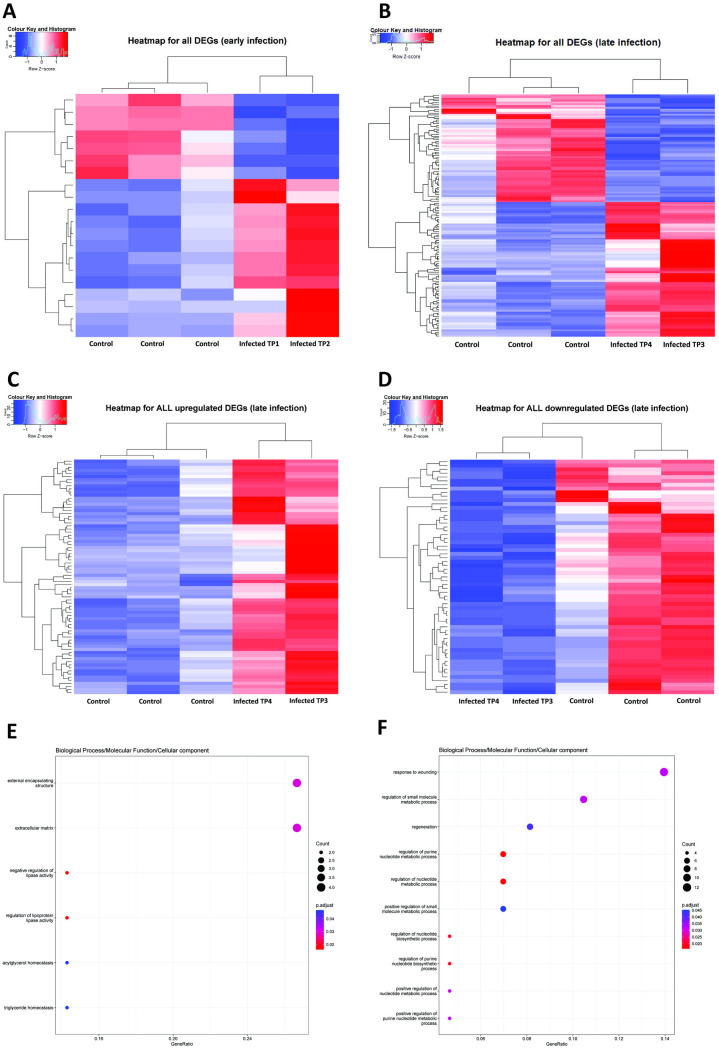
Enriched gene ontology associated with transcriptional response in liver during early and late response to infection. (A – D) Heatmaps of all significant differentially expressed genes in the liver (early and late response to infection). (A) Heatmap of all genes differentially expressed during early response to infection, (B) Heatmap of all genes differentially expressed during late response to infection, (C) Heatmap of genes upregulated during late response to infection, (d) Heatmap of genes downregulated during late response to infection. (E and F) Gene Ontology (GO) enrichment analysis of genes differentially expressed in the liver during early and late phase of infection.TP means timepoint.

The GO enrichment analysis of the liver transcriptome during the early immune response to infection showed that the regulation of cell structure (e.g., ANGPTL3, ANGPTL4, ELFN2, COL5A3), including the external encapsulating structure and extracellular matrix, has the highest gene ratio with significantly expressed genes. Conversely, genes responsible for regulating acylglycerol homeostasis (e.g., ANGPTL3, ANGPTL4) and triglyceride homeostasis (e.g., ANGPTL3, ANGPTL4) were found to be transcriptionally downregulated (Fig 10E). It is worth noting that genes relating to innate immune response to viral infection (e.g., DHX58, MX1, IFITM-like), cell structure integrity (e.g., ANGPTL3, ANGPTL4, ELFN2, COL5A3) and transcription factors (e.g., TUB) remained upregulated throughout the acute phase of infection (1–6 dpi) (Fig 11A).

During the late response to infection, as shown by the GO analysis, the top three categories with the most significantly expressed genes were response to wounding, small molecule metabolic process, and regeneration (Fig 11F). Genes with antiviral activities, including T cell proliferation and interferon pathways (e.g., IFITM-like, MX1, DHX58, IFI27L2A, LY6E-like, PARP9) were highly transcribed in 7–9 dpi, but it appears that such high level of transcription were not sustained into the fourth stage of infection (11–21 dpi) (Fig 11B). It is worth noting that genes relating to hypoxia, inflammation and regulation of liver physiological processes were upregulated throughout the third and fourth phase of infection (7–21 dpi) (Fig 11B).

### Transcription profile of selected cytokine genes in infected animals

Key canonical inflammatory, antiviral, cell proliferation and apoptosis genes were not among the significantly differentially expressed by RNAseq-based transcriptomic analysis. Therefore, we further investigated the transcription profiles of selected inflammatory (*Il-1 β* like, *Il-34*, *Hif-1-α*, *Csf1[M-CSF]* and *tgf*-*β*), cell proliferation and apoptosis (*Ki67* and *Casp9*) and antiviral (*Irf3*, *Oasl*, *Mx1*-like and *Tfeb*) cytokine genes by RT-qPCR. We compared the transcription profiles in the liver, spleen, and kidney at specific time points. The time points were chosen based on the viremia kinetics, viral loads and histopathology in the mentioned tissues (Fig 5). Specifically, we compared day 1, 2, and 3 dpi with 7, 9, 15, and 21 dpi. Additionally, we compared day 1, 2, 3 dpi with each other. The comparisons and their statistical significances are summarized in S11, S12 and S13 Tables.

Upregulation of most antiviral cytokine genes (*Irf3*, *Oasl* and *Tfeb*) in the liver and kidney peaked at 7–9 dpi; while the level of upregulation remained constant across all timepoints in the spleen (S4B Fig and S11 Table). The expression of pro- and anti-inflammatory and antiviral cytokine genes was intermittently upregulated in kidneys as compared to uninfected (sham infected) controls. Upregulation of all pro- and anti-inflammatory and antiviral cytokine genes peaked at 7 and 9 dpi except *Mx1*-like gene (S2 and S7C Figs). A similar trend was observed for the detection of viral genome in kidneys (Fig 2B). A similar trend was observed for *Hif1-α*, *Il-1 β*-like and *Tgf-β* in liver. In contrast, *Il-34* and *Csf1[M-CSF]* transcripts were progressively upregulated between 1 and 7 dpi. While they remained upregulated, a decline in expression was observed from 9 dpi until the completion of the experiment (21 dpi) (S7 Fig).

Overall, for all three groups of genes (inflammatory, cell proliferation and apoptosis, and antiviral), the transcriptional profile is divided into three main phases except for the *Mx1* like gene (S4, S5 and S6 Figs). The initial phase, probably corresponding to the incubation period, runs between 1 dpi and 2 dpi. During this phase, most cytokine and transcription factor genes remain at the baseline expression level (equal to uninfected). A few cytokine genes were upregulated at low amplitude during this phase (S4, S5 and S6 Figs). Also, the pattern and level of expression was tissue/organ dependent. For instance, mRNA expression of *Ki67, Casp9, Tfeb* and **Tgf*β* was constant in spleen while it was intermittently upregulated in liver and kidney (S4, S5 and S6 Figs). The transcription of cytokine and transcription factor genes were upregulated from 3-4 dpi to 15 dpi. The peak of cytokine and transcription factor gene expression was observed between 7 and 9 dpi. The peak and plateau phases overlapped, possibly because from 7 dpi animals were sampled at two-day intervals. Hence, the plateau phase could have been missed for some cytokine and transcription factor genes.

## Discussion

In this study we extended our previous studies of WNV infection in hatchling saltwater crocodiles [8,18] demonstrating that the main sources of virus excreted via the cloaca into the water, leading to fecal-oral transmission, are liver, pancreas, kidney and cloaca. Initial virus replication, following challenge by injection, may take place in the spleen with viremic spread to other parenchymal organs and skin, although if the infection route is oral, initial replication likely takes place in the gastrointestinal tract [26].

As was reported previously [8], none of the infected saltwater crocodiles developed clinical disease, and only a few (4/69, i.e., 6%) of the animals developed grossly detectable skin lesions during the 21 days study period. However, our previous studies, as well as experience from studies of alligators, suggest lesions may develop over a period of up to three months post-infection [8,27,28]. Therefore, the relatively low incidence of lesions in this study was anticipated, since animals were sequentially terminated, and only four WNV–challenged animals plus five in–contact controls remained at the last termination time point 21 dpi. Hence, we likely abrogated lesion development in several of the animals terminated earlier during infection. This contention is supported by the finding that 13 animals, terminated at earlier timepoints and with grossly normal skin, had lymphohistiocytic dermatitis, detected microscopically in skin samples randomly collected from the abdominal region. These cellular infiltrates could be interpreted as precursors to pix development. In this study, only the pancreas showed a direct association between small aggregates of lymphohistiocytic cell infiltrates and presence of viral NS1 protein, as detected by IHC. Our previous investigations have shown the presence of WNV RNA but not replicating virus nor viral antigen in the lymphoid aggregates of skin lesions [8,9,27], suggesting that the viral replication in the skin may be abrogated. The phenotype of the immune cells in those infiltrates has yet to be established for the crocodile but are likely to be similar to those detected in American alligators (*Alligator mississippiensis*) [29]. Whether those lymphoid cells would be exerting antiviral activity or are there as a bystander phenomenon remains to be elucidated, although the former seems likely.

Given the sustained viremia, despite an emerging neutralizing antibody response starting around 9 dpi, it is likely that viral replication continues in some of the parenchymal organs past 21 dpi [8]. It remains to be determined for how long this may continue and where the virus might persist. However, given that the transcriptomic analysis suggests that T lymphocyte activation is a relatively late-onset phenomenon and that antibodies might be insufficient to clear the infection, this is clearly an important aspect to explore from both a farming management (including vaccination schedules), a public health perspective, for the general understanding of reptilian immune defenses and the pathogenesis of WNV infection.

The transcriptomic analysis of two of the organs involved in WNV replication, the kidney and liver, showed a predominance of changes in cellular metabolism, characterized by increased transcription of ribosomal and mitochondrial structural and metabolic pathways. This finding corroborates earlier studies on flaviviruses manipulating mitochondrial metabolism to evade antiviral innate immunity [30]. Early transcriptional upregulation of inflammatory, antiviral (e.g., interferon-stimulated genes such as *Ifi27l2, Ifi27l2a, Ifit5, Cxcl10-like, Trim27l, Herc5, Trim25l, Samd9l, Dhx58*) and apoptotic pathways were also evident, while transcripts relating to adaptive immune responses only appeared relatively late (Figs 7 – 10 and S5, S6, S9 and S10 Tables).

The immune responses of reptiles to infectious agents are still poorly characterized, and direct comparisons between reptiles and mammals are rare. However, a recent report comparing the response of lizards and mice to *Leishmania* spp. infection revealed a predominantly metabolic response in the resistant lizards compared to the susceptible mice [31]. Other reports have demonstrated that ambient temperatures influence how reptiles respond to infections [32], and that responses are generally slow to develop and potentially counterintuitive when compared to mammalian species [33]. American alligators are exquisitely susceptible to WNV and may develop neurological and gastrointestinal signs of disease in addition to skin lesions [28]. Therefore, it would be of interest to directly compare the responses of saltwater crocodiles and alligators to WNV strains of varying virulence. This comparison could help assess their response profiles and determine whether the observed differences in susceptibility are due to the host, the virus, or a combination of both factors. While the WNV_KUN_ strain utilized in this study is considered the most virulent strain currently circulating in Australia, it is noted that it is less virulent in mice than the WNV strains circulating in North America [34]. In this context it is also of note that some genes involved in inflammatory processes were downregulated in the saltwater crocodiles following WNV infection, including *serping1* (Serpin Family G Member 1), which promotes inflammation via the complement cascade [35,36], *nr1d1* (nuclear receptor subfamily 1 group D member 1) which regulates genes that function in metabolic, inflammatory and cardiovascular processes, and *rassf7* (Ras association domain family member 7) which is involved in apoptotic process and signal transduction (S5-S7 Tables). It could be hypothesized that, upon infection, saltwater crocodiles may control the severity of the disease through the downregulation of some pro-inflammatory genes that would lead to tissue destruction and pathology, as has also been described for bats [37].

Since some of the principal, canonical anti-inflammatory, cellular proliferation, apoptosis and antiviral transcripts did not appear among the significantly differentially expressed genes in the RNAseq-based analysis, we sought to examine the transcriptional profile of a range of such genes using RT-qPCR. Our focus was on WNV-targeting antiviral effector genes and ISGs (*Irf3, Oasl,* and *Mx1*), pro- and anti-inflammatory genes (**il1*β, *il34, hif-1*α, *csf-1[m-csf],** and **tgf*β*), as well as cell proliferation and apoptosis (*ki67* and *casp 9*) genes. Overall, we observed a statistically significant upregulation of transcription of most of these genes (mainly antiviral and anti-inflammatory) at 7 and 9 dpi in liver and kidney tissues (S3-S7 Figs).

The apparent discrepancy between the RNAseq-based analysis showing downregulation of a range of pro-inflammatory genes, while the RT-qPCR analysis suggests increased transcription of at least a subset of pro-inflammatory molecules may be reconciled by proportionality of cell type contributions in the two sample sets. Transcripts for cell metabolism, etc., from the parenchymal cells, such as hepatocytes and kidney tubular epithelial cells, would dominate in the RNAseq readout, while the targeting of specific transcripts by RT-qPCR would exclude all such transcripts. Hence, it is possible to have ongoing overall systemic anti-inflammatory processes, while minimal and very localized inflammation, in the form of lymphohistiocytic cell infiltrates, develops in virus-infected organs.

## Conclusion

In the present study we demonstrated that WNV primarily replicates in the spleen, liver, pancreas, and kidney, with virus also present in the cloaca and the third eyelid. There also appeared to be a relationship between viral genome detection in kidneys and viral genome detection in cloacal swabs in all infection phases, suggestive of a key role for the kidney in virus shedding. However, it seems highly likely that the gastrointestinal tract, including pancreas and liver, also contributes to the fecal-oral route of transmission.

The transcriptomic investigations suggest that saltwater crocodiles respond to virus infection with primarily metabolic changes that may limit viral replication. Additional innate immune responses, including antiviral cytokines and regulation of apoptosis pathways may add another level of restriction on viral replication and dissemination within the animal. At the same time, such mechanisms may also allow the virus to persist longer in some nidi, as has been seen in a hamster model of WNV infection [38,39], thereby resulting in a protracted antibody response.

The RNAseq results of our study need to be interpreted with the following limitations in mind. This is the first study that holistically investigates interaction between a crocodilian species and WNV, particularly, looking at the immune response at different stages of infection. However, due to the lack of fully characterized GO of any reptilian species, we performed the GO analysis referring to the human genome. Also, several crocodilian genes do not have gene annotation or gene ontology annotation. Therefore, while this study provides insights into the pathophysiological responses to experimental WNV infection in saltwater crocodiles, further studies are warranted to elucidate the interaction between WNV and the host. Our study could serve as a baseline to further study the crocodilian interaction with WNV and other pathogens.

## Supporting information

S1 Table Schedule of crocodile culling and sample collection. (DOCX)

S2 Table Comparison of virus titers recovered by RT-qPCR vs. virus isolation by cell culture in a selected set of samples. (DOCX)

S3 Table Summary viraemia proportion by qRT-PCR at different time points. (DOCX)

S4 Table Gene ontology categories in the kidney during late – stage response infection. (DOCX)

S5 Table Genes expressed in kidney during early response to infection. (DOCX)

S6 Table Genes expressed in kidney during late response to infection. (DOCX)

S7 Table Gene ontology categories in the liver during early – stage response infection. (DOCX)

S8 Table Gene ontology categories in the liver during late – stage response infection. (DOCX)

S9 Table Genes expressed in liver during early response to infection. (DOCX)

S10 Table Genes expressed in liver during late response to infection. (DOCX)

S11 Table Summary of p-values from Tukey’s multiple comparisons test of the level of upregulated liver cytokine and transcription factor expression between the different timepoints^£.^ (DOCX)

S12 Table Summary of p-values from Tukey’s multiple comparisons test of the level of upregulated splenic cytokine and transcription factor expression between the different timepoints^£.^ (DOCX)

S13 Table Summary of p-values from Tukey’s multiple comparisons test of the level of upregulated kidney cytokine and transcription factor expression between the different timepoints^£.^ (DOCX)

S1 Fig Distinct transcriptional signatures in the kidney and liver during early and late response to infection. (A and B) MA-plot for early and late response to infection in kidney. (C and D). MA-plot for early and late response to infection in liver.
(TIF)

S2 Fig Antiviral cytokine and transcription factor gene transcriptional profile in liver, spleen, and kidney at various timepoints. The discontinuous line represents baseline transcription (one-fold-change equivalent to cytokine gene expression in mock-infected animal). The statistical comparison of the transcriptional profile of individual cytokine and transcription factor genes at different time points in various tissues was tested by a two-way ANOVA carried out for multiple comparison analysis with the α-level set at 0.05 with a Tukey’s post-test, with individual variances computed for each comparison. To compare the overall transcriptional profile of cytokine and transcription factor genes in tissues, ordinary two-way ANOVA with a Tukey’s multiple comparisons test, with a single pooled variance.
(TIF)

S3 Fig Pro- and anti-inflammatory cytokine gene transcriptional profile in liver, spleen, and kidney tissues at various timepoints. The discontinuous line represents baseline transcription (one-fold change equivalent to cytokine gene expression in mock infected animal). The statistical comparison of the transcriptional profile of individual cytokine genes at different time points in various tissues was tested by a two-way ANOVA carried out for multiple comparison analysis with the α-level set at 0.05 with a Tukey’s post-test, with individual variances computed for each comparison. To compare the overall transcriptional profile of cytokine genes in tissues, ordinary two-way ANOVA with a Tukey’s multiple comparisons test, with a single pooled variance.
(TIF)

S4 Fig Cell proliferation and apoptosis cytokine gene transcriptional profile in liver, spleen, and kidney tissues at various timepoints. The discontinuous line represents baseline transcription (one-fold change equivalent to cytokine gene expression in mock-infected animal). The statistical comparison of the transcriptional profile of individual cytokine genes at different time points in various tissues was tested by a two-way ANOVA carried out for multiple comparison analysis with the α-level set at 0.05 with a Tukey’s post-test, with individual variances computed for each comparison. To compare the overall transcriptional profile of cytokine genes in tissues, ordinary two-way ANOVA with a Tukey’s multiple comparisons test, with a single pooled variance.
(TIF)

S5 Fig Comparison of antiviral cytokine and transcription factor gene transcriptional kinetics in liver, spleen, and kidney at various time points post-infection. A discontinuous line at log10 (Y + 1) represents baseline transcriptional profile (one-fold change equivalent to cytokine and transcription factor gene expression in mock-infected animals). The Tukey’s multiple comparisons test was performed to compare median of antiviral cytokine and transcription factor gene expression at various time points in each tissue. Significant statistical difference thresholds are *p ≤ 0.05, **p ≤ 0.01, ***p ≤ 0.001, ****p ≤ 0.0001, ns = not significant.
(TIF)

S6 Fig Cell proliferation and apoptosis cytokine gene transcriptional profile in liver, spleen, and kidney at various timepoints post infection. A discontinuous line at log10 (Y + 1) represents baseline transcriptional profile (one-fold change equivalent to cytokine gene expression in mock-infected animal). The Tukey’s multiple comparisons test was performed to compare median of cell proliferation and apoptosis cytokine gene expression at various time points in each tissue. Significant statistical difference thresholds are *p ≤ 0.05, **p ≤ 0.01, ***p ≤ 0.001, ****p ≤ 0.0001, ns = not significant.
(TIF)

S7 Fig Pro-inflammatory and anti-inflammatory cytokine gene transcriptional profile in liver, spleen, and kidney at various timepoints post infection. A discontinuous line at log10 (Y + 1) represents baseline transcriptional profile (one-fold change equivalent to cytokine gene expression in mock infected animals). The Tukey’s multiple comparisons test was performed to compare median of cell proliferation and apoptosis cytokine gene expression at various time points in each tissue. Significant statistical difference thresholds are *p ≤ 0.05, **p ≤ 0.01, ***p ≤ 0.001, ****p ≤ 0.0001, ns = not significant.
(TIF)
