# Supplementary material for: Tissue tropism, pathology, and pathogenesis of West Nile virus infection in saltwater crocodile (Crocodylus porosus)
Source: PLoS Negl Trop Dis. 2025 Aug 4;19(8):e0013385. doi: 10.1371/journal.pntd.0013385 (PMC12331170; doi:10.1371/journal.pntd.0013385)
Supplement: S1 Table — (DOCX) [file pntd.0013385.s001.docx]

**S1 Table.** Schedule of crocodile culling and sample collection

| **Timepoint (dpi)** | **Number of terminated animals** | | | |
| --- | --- | --- | --- | --- |
|  | **Infected** | **Control** | **in-pen control** | **Total** |
| 1 | 5 | 2 | 0 | 7 |
| 2 | 5 | 0 | 0 | 5 |
| 3 | 5 | 0 | 0 | 5 |
| 4 | 5 | 0 | 4 | 9 |
| 5 | 5 | 0 | 0 | 5 |
| 6 | 5 | 0 | 0 | 5 |
| 7 | 5 | 2 | 0 | 7 |
| 9 | 5 | 0 | 4 | 9 |
| 11 | 5 | 0 | 0 | 5 |
| 13 | 5 | 2 | 4 | 11 |
| 15 | 5 | 0 | 0 | 5 |
| 17 | 5 | 0 | 4 | 9 |
| 19 | 5 | 2 | 0 | 7 |
| 21 | 4 | 0 | 6 | 9 |
