# Supplementary material for: Tissue tropism, pathology, and pathogenesis of West Nile virus infection in saltwater crocodile (Crocodylus porosus)
Source: PLoS Negl Trop Dis. 2025 Aug 4;19(8):e0013385. doi: 10.1371/journal.pntd.0013385 (PMC12331170; doi:10.1371/journal.pntd.0013385)
Supplement: S2 Table — (DOCX) [file pntd.0013385.s002.docx]

**S2 Table.** Comparison of virus titers recovered by RT-qPCR vs. virus isolation by cell culture in a selected set of samples

| **Day post infection** | **Tag #** | **Treat. Group** | **Samples** | **Virus titer in various tissue samples** | |
| --- | --- | --- | --- | --- | --- |
|  |  |  |  | **RT-qPCR titre (log_10_ TCID_50_ equivalent/g)^*^** | **Virus isolation titer (log_10_ TCID_50_/g)^#^** |
| Day 3 | H01 | Infected | Liver | 3.31 | BLD |
|  |  |  | Spleen | 3.74 | 3.80 |
|  | H40 | Infected | Liver | 3.52 | 3.80 |
|  |  |  | Kidney | 3.57 | BLD |
|  |  |  | Cloaca | 2.71 | BLD |
|  | H38 | Infected | Liver | 1.42 | 3.80 |
|  |  |  | Spleen | 2.61 | 3.80 |
|  |  |  | Kidney | 1.86 | BLD |
|  |  |  | Cloaca | 1.04 | BLD |
| Day 4 | G13 | Infected | Spleen | 0.53 | 3.80 |
|  |  |  | Kidney | 0.97 | BLD |
|  |  |  | Cloaca | 2.24 | BLD |
| Day 6 | G23 | Infected | Liver | 2.61 | 3.80 |
|  |  |  | Spleen | 2.65 | BLD |
|  |  |  | Kidney | 2.04 | BLD |
|  | G25 | Infected | Liver | 2.27 | 3.80 |
|  |  |  | Spleen | 3.16 | BLD |
| Day 7 | H17 | Infected | Liver | 1.20 | BLD |
|  |  |  | Spleen | 1.16 | BLD |
|  |  |  | Kidney | 1.52 | BLD |
|  |  |  | Cloaca | 4.03 | 4.30 |

BLD: below limit of detection, **^*^**limit of detection: 0.53 log_10_ TCID_50_ equivalent/g, **^#^**limit of detection: 3.80 log_10_ TCID_50_ equivalent/g.
