# Supplementary material for: Tissue tropism, pathology, and pathogenesis of West Nile virus infection in saltwater crocodile (Crocodylus porosus)
Source: PLoS Negl Trop Dis. 2025 Aug 4;19(8):e0013385. doi: 10.1371/journal.pntd.0013385 (PMC12331170; doi:10.1371/journal.pntd.0013385)
Supplement: S3 Table — (DOCX) [file pntd.0013385.s003.docx]

**S3 Table.** Summary viraemia proportion by qRT-PCR at different time points

| **Day post infection** | **Animal ID #** | **Treat. Group** | **Viraemia (TCID_50_ equivalent/mL)** | **Average viraemia titer (TCID_50_ equivalent/mL)** |
| --- | --- | --- | --- | --- |
| 1 dpi | G42 | Infected | 2.56 | 2.70 |
|  | H14 | Infected | 3.35 |  |
|  | H07 | Infected | 3.90 |  |
|  | G24 | Infected | 3.71 |  |
| 2 dpi | G12 | Infected | 1.38 | 2.07 |
|  | G15 | Infected | 2.92 |  |
|  | G19 | Infected | 0.88 |  |
|  | G26 | Infected | 2.81 |  |
|  | G10 | Infected | 2.35 |  |
| 3 dpi | H01 | Infected | 4.59 | 3.81 |
|  | H44 | Infected | 3.27 |  |
|  | G49 | Infected | 3.06 |  |
|  | H40 | Infected | 4.28 |  |
|  | H38 | Infected | 3.84 |  |
| 4 dpi | H47 | Infected | 3.17 | 2.21 |
|  | G16 | Infected | 1.94 |  |
|  | G13 | Infected | 3.43 |  |
|  | G20 | Infected | 2.50 |  |
| 5 dpi | H20 | Infected | 1.91 | 2.37 |
|  | H29 | Infected | 4.44 |  |
|  | H33 | Infected | 1.10 |  |
|  | H08 | Infected | 4.42 |  |
| 6 dpi | G23 | Infected | 3.74 | 1.86 |
|  | G17 | Infected | 3.00 |  |
|  | G25 | Infected | 2.56 |  |
| 7 dpi | H17 | Infected | 2.17 | 1.02 |
|  | G36 | Infected | 0.91 |  |
|  | H30 | Infected | 2.02 |  |
| 9 dpi | H26 | Infected | 2.36 | 2.36 |
| 11 dpi | H28 | Infected | 0.81 | 0.81 |
| 13 dpi | H15 | Infected | 1.06 | 1.06 |
| 15 dpi | H19 | Infected | 0.88 | 0.88 |
| 21 dpi | H25 | in-pen control | 1.01 | 1.01 |
