# Supplementary material for: Tissue tropism, pathology, and pathogenesis of West Nile virus infection in saltwater crocodile (Crocodylus porosus)
Source: PLoS Negl Trop Dis. 2025 Aug 4;19(8):e0013385. doi: 10.1371/journal.pntd.0013385 (PMC12331170; doi:10.1371/journal.pntd.0013385)
Supplement: S4 Table — (DOCX) [file pntd.0013385.s004.docx]

**S4 Table.** Gene ontology categories in the kidney during late – stage response infection

| **Description** | **GeneRatio** | **BgRatio** | **p value** | **p. adjust** | **q value** | **Gene ID** | **Count** |
| --- | --- | --- | --- | --- | --- | --- | --- |
| Ribosomal subunit | 61/2485 | 140/10963 | 2.71E-08 | 1.68E-05 | 1.60E-05 | RPL11, RPL10A, RPS15, RPS27L, RPS3A, RPL18A, RPL32, RPL15, RPL26L1, RPS14, RPS21, RPL31, RPS12, RPL35A, MRPS24, DAP3, MRPS6, RPL19, MRPL48, MTERF4, RPL3, RPSA, RPS20, MRPL54, RPL8, RPS16, MRPS18A, RPLP1, RPL13A, UBA52, RPS19, MRPS35, MRPL32, MRPL41, MRPS28, MRPL43, RPL37, MRPL36, RPL24, MRPL15, MRPL39, MRPS10, RPL9, RPLP0, MRPS26, ZCCHC17, MRPS33, MRPL37, MRPL46, MRPL51, RPS7, RPL34, MRPL50, MRPS31, NSUN4, MRPS14, MRPL40, MRPS18C, RPL14, AURKAIP1, MRPL58 | 61 |
| Ribosome | 68/2485 | 166/10963 | 8.62E-08 | 2.67E-05 | 2.54E-05 | RPL11, LARP1, RPL10A, RPS15, RPS27L, RPS3A, NUFIP2, RPL18A, RPL32, RPL22L1, RPL15, RPL26L1, RPS14, RPS21, RPL31, RPS12, RPL35A, MRPS24, DAP3, MRPS6, RPL19, MRPL48, MTERF4, RPL3, RPSA, RPS20, MRPL54, RPL8, RPS16, MRPS18A, RPLP1, RPL13A, UBA52, RPS19, SERP1, MRPS35, MRPL32, MRPL41, MRPS28, MRPL43, RPL37, MRPL36, RPL24, MRPL15, MRPL39, MRPS10, RPL9, RPLP0, MRPS26, ZCCHC17, MRPS33, MRPL37, MRPL46, EIF3H, MRPL51, RPS7, RSL24D1, RPL34, MRPL50, MRPS31, NSUN4, MRPS14, MRPL40, MRPS18C, RPL14, AURKAIP1, MRPL58, DNAJC21 | 68 |
| Large ribosomal subunit | 38/2485 | 86/10963 | 7.39E-06 | 0.00152709 | 0.00144931 | RPL11, RPL10A, RPL18A, RPL32, RPL15, RPL26L1, RPL31, RPL35A, RPL19, MRPL48, MTERF4, RPL3, MRPL54, RPL8, MRPS18A, RPLP1, RPL13A, UBA52, MRPL32, MRPL41, MRPL43, RPL37, MRPL36, RPL24, MRPL15, MRPL39, RPL9, RPLP0, ZCCHC17, MRPL37, MRPL46, MRPL51, RPL34, MRPL50, NSUN4, MRPL40, RPL14, MRPL58 | 38 |
| Cytosolic ribosome | 32/2485 | 70/10963 | 1.64E-05 | 0.00253971 | 0.00241035 | RPL11, RPL10A, RPS15, RPS27L, RPS3A, RPL18A, RPL32, RPL15, RPL26L1, RPS14, RPS21, RPL31, RPS12, RPL35A, RPL19, RPL3, RPSA, RPS20, RPL8, RPS16, RPLP1, RPL13A, UBA52, RPS19, RPL37, RPL24, RPL9, RPLP0, ZCCHC17, RPS7, RPL34, RPL14 | 32 |
| Cytosolic large ribosomal subunit | 21/2485 | 42/10963 | 9.40E-05 | 0.01165042 | 0.01105702 | RPL11, RPL10A, RPL18A, RPL32, RPL15, RPL26L1, RPL31, RPL35A, RPL19, RPL3, RPL8, RPLP1, RPL13A, UBA52, RPL37, RPL24, RPL9, RPLP0, ZCCHC17, RPL34, RPL14 | 21 |
| Mitochondrial protein-containing complex | 65/2485 | 192/10963 | 0.00023465 | 0.02424707 | 0.02301208 | DNAJC15, MPC1, MICU3, TIMM13, TIMM8A, NDUFS4, NDUFV2, DNAJC19, NDUFA4, MRPS24, DAP3, MRPS6, NDUFB5, MRPL48, SUCLG2, MTERF4, NDUFA8, MRPL54, MRPS18A, TOMM6, NDUFA6, TOMM20, NDUFB8, MRPS35, MRPL32, MRPL41, MRPS28, MPC2, TIMM17A, MRPL43, MRPL36, SLC25A6, MRPL15, MRPL39, MRPS10, NDUFC1, NDUFB1, APOO, PPIF, NDUFB9, VDAC1, MFN1, MRPS26, NDUFB4, MTX2, MRPS33, TIMM21, MRPL37, MRPL46, MRPL51, POLG, NDUFA2, MRPL50, MRPS31, NSUN4, MRPS14, MRPL40, MRPS18C, SMDT1, AURKAIP1, NDUFB2, IMMP2L, TIMM22, TIMM9, MRPL58 | 65 |
| Small ribosomal subunit | 25/2485 | 57/10963 | 0.0003017 | 0.02672197 | 0.02536092 | RPS15, RPS27L, RPS3A, RPS14, RPS21, RPS12, MRPS24, DAP3, MRPS6, RPSA, RPS20, RPS16, MRPS18A, UBA52, RPS19, MRPS35, MRPS28, MRPS10, MRPS26, MRPS33, RPS7, MRPS31, MRPS14, MRPS18C, AURKAIP1 | 25 |
| Cortical cytoskeleton | 34/2485 | 87/10963 | 0.00040768 | 0.02875177 | 0.02728734 | SPTBN2, NUMA1, SHROOM3, ERC1, SHROOM2, CALD1, MAEA, SPTBN1, EPB42, CDH1, RIMS1, AKAP13, SPTAN1, MYH9, LLGL1, EEF1A1, FLOT2, FCHSD1, PLS1, PCLO, DBN1, LLGL2, BSN, TRPV4, EPB41, PPP1R9B, CDH2, CLDN5, MYADM, PVALB, DMTN, MISP, CIB2, ANLN | 34 |
| Cortical actin cytoskeleton | 25/2485 | 58/10963 | 0.00041736 | 0.02875177 | 0.02728734 | SPTBN2, SHROOM3, SHROOM2, CALD1, MAEA, SPTBN1, CDH1, AKAP13, SPTAN1, MYH9, LLGL1, EEF1A1, FLOT2, FCHSD1, PLS1, LLGL2, TRPV4, PPP1R9B, CDH2, CLDN5, MYADM, PVALB, MISP, CIB2, ANLN | 25 |
| Organellar ribosome | 29/2485 | 73/10963 | 0.00077437 | 0.04364653 | 0.04142345 | MRPS24, DAP3, MRPS6, MRPL48, MTERF4, MRPL54, MRPS18A, MRPS35, MRPL32, MRPL41, MRPS28, MRPL43, MRPL36, MRPL15, MRPL39, MRPS10, MRPS26, MRPS33, MRPL37, MRPL46, MRPL51, MRPL50, MRPS31, NSUN4, MRPS14, MRPL40, MRPS18C, AURKAIP1, MRPL58 | 29 |
| Mitochondrial ribosome | 29/2485 | 73/10963 | 0.00077437 | 0.04364653 | 0.04142345 | MRPS24, DAP3, MRPS6, MRPL48, MTERF4, MRPL54, MRPS18A, MRPS35, MRPL32, MRPL41, MRPS28, MRPL43, MRPL36, MRPL15, MRPL39, MRPS10, MRPS26, MRPS33, MRPL37, MRPL46, MRPL51, MRPL50, MRPS31, NSUN4, MRPS14, MRPL40, MRPS18C, AURKAIP1, MRPL58 | 29 |
| cytoplasmic stress granule | 26/2485 | 64/10963 | 0.00096074 | 0.04963829 | 0.04711002 | LARP1, EIF4G1, NUFIP2, HIPK2, PRRC2C, EIF4E, GRB7, MBNL1, GIGYF2, UBAP2L, TRIM25, TIAL1, MCRIP2, DDX6, RC3H1, RPTOR, YBX1, ATXN2, CASC3, PRKAA2, LARP4, CIRBP, ZFAND1, DYRK3, IGF2BP1, DDX25 | 26 |
| Structural constituent of ribosome | 51/2460 | 124/10811 | 3.37E-06 | 0.00171107 | 0.00162923 | RPL11, RPL10A, RPS15, RPS27L, RPS3A, RPL18A, RPL32, RPL22L1, RPL15, RPL26L1, RPS14, RPS21, RPL31, RPS12, RPL35A, MRPS24, DAP3, MRPS6, RPL19, RPL3, RPSA, RPS20, MRPL54, RPL8, RPS16, MRPS18A, RPLP1, RPL13A, UBA52, RPS19, MRPS35, MRPL32, MRPL41, MRPL43, RPL37, MRPL36, RPL24, MRPL15, RPL9, RPLP0, MRPS33, MRPL37, MRPL46, MRPL51, RPS7, RSL24D1, RPL34, MRPS31, MRPS14, MRPS18C, RPL14 | 51 |
| Transcription coregulator activity | 118/2460 | 359/10811 | 5.23E-06 | 0.00171107 | 0.00162923 | KMT2D, SPEN, NSD1, NCOA1, PFDN5, BCL9L, NCOR2, CITED2, EP300, ATF7IP, TACC1, KAT6A, ARID1A, YAF2, RERE, NCOR1, HIPK2, NUP98, MED10, BCL9, MED12, HMGB1, SUFU, MED30, ZMIZ2, NOTCH1, RBFOX2, MED13, GON4L, KDM5B, MED4, ABL1, KDM3B, ATN1, NUCKS1, TRIM8, MUC1, EZH2, ASXL1, COPS5, TRIM25, TOB2, KMT2C, TFAP2A, LDB1, AEBP2, CALCOCO1, TLE3, MED21, PHF12, MAML2, SIN3A, URI1, THRAP3, MED20, HSBP1, SMARCC1, SUB1, EWSR1, KDM2A, RNF20, BCORL1, JUP, CCND1, PPRC1, PARK7, CBFB, FHL3, NCOA5, KMT5A, SMARCA2, MTDH, PPARGC1B, MED1, KAT2B, ZFPM1, TAF11, SETD3, RAP2C, TP53BP1, MAML3, PIAS1, NRIP1, KDM7A, TCF25, TRIM32, SRSF2, TRERF1, GMNN, MYBBP1A, KDM3A, KDM5A, CENPJ, ING4, TBL1X, DTX1, SFMBT2, PARP9, SMARCE1, NOC2L, HELZ2, VGLL1, USP16, HMGA2, C1D, PIAS4, PIR, ENY2, MTA3, TAF15, MIER3, CBFA2T3, MED6, FHL2, SRA1, MIER2, DNMT3B, TAF5L | 118 |
| Transcription factor binding | 141/2460 | 444/10811 | 5.32E-06 | 0.00171107 | 0.00162923 | SPEN, CTDP1, NSD1, NCOA1, NFYB, PAX2, NCOR2, CITED2, EP300, TACC1, KAT6A, ARID1A, ATF7, NCOR1, SREBF1, BAZ2A, HIPK2, MED12, HMGB1, SUFU, NFAT5, MED30, RBFOX2, THRA, VDR, MED13, FOXO4, EIF4E, CHD4, VHL, PBX1, MED4, CNOT1, SP1, ATF2, NUCKS1, PURB, TAF1, EZH2, ASXL1, TOB2, PPARG, LDB1, RBX1, PHF12, HNF4A, RARA, TBX2, SIN3A, THRAP3, ZNF618, BAIAP2, MTOR, NOTCH2, FOXP1, CRTC1, RPTOR, SUMO1, ZBTB7A, NR1D1, DDX5, COMMD6, JUP, TFDP1, MED25, PPRC1, PARK7, ZNF644, UBXN7, SRC, CRTC2, MTDH, SOX9, PPARGC1B, MED1, KAT2B, NEK6, ZFPM1, TAF11, SETD3, TP53BP1, HMGN3, TEAD3, PIAS1, EHMT1, NRIP1, ETS2, PSMD10, PRDM5, NCAPG2, MDFIC, TRIM32, BDP1, TCF3, TRERF1, GATA3, LATS1, STK4, ZNF516, GMNN, STK36, EPAS1, KDM3A, TERT, MEF2D, TCF12, TTC8, PTPN2, EGR2, TAF10, MAD2L2, ESRRB, TBL1X, PARP1, COMMD7, PARP9, SMARCE1, NOC2L, MAX, NR4A1, ZNF703, SPI1, PROX1, HMGA2, C1D, BCL6, AR, METTL23, NR1H4, ESR1, BBS5, MED6, PASD1, FHL2, TFDP2, HEY1, FLT3, EED, MLXIPL, TP73, NR5A1 | 141 |
| DNA-binding transcription factor binding | 116/2460 | 354/10811 | 7.38E-06 | 0.00177768 | 0.00169266 | SPEN, CTDP1, NSD1, NCOA1, NFYB, NCOR2, CITED2, EP300, TACC1, KAT6A, ARID1A, NCOR1, BAZ2A, HIPK2, MED12, HMGB1, MED30, RBFOX2, VDR, MED13, FOXO4, EIF4E, CHD4, VHL, PBX1, MED4, CNOT1, SP1, ATF2, NUCKS1, PURB, TAF1, ASXL1, TOB2, PPARG, LDB1, RBX1, HNF4A, TBX2, SIN3A, THRAP3, NOTCH2, FOXP1, CRTC1, ZBTB7A, DDX5, COMMD6, JUP, TFDP1, MED25, PARK7, UBXN7, SRC, CRTC2, MTDH, SOX9, PPARGC1B, MED1, KAT2B, NEK6, ZFPM1, TAF11, SETD3, TP53BP1, HMGN3, TEAD3, PIAS1, NRIP1, ETS2, PSMD10, PRDM5, NCAPG2, MDFIC, TRIM32, TCF3, TRERF1, GATA3, LATS1, STK4, ZNF516, GMNN, EPAS1, KDM3A, MEF2D, TCF12, TTC8, PTPN2, EGR2, TAF10, MAD2L2, ESRRB, TBL1X, PARP1, COMMD7, PARP9, SMARCE1, NOC2L, MAX, NR4A1, ZNF703, SPI1, PROX1, HMGA2, C1D, BCL6, AR, METTL23, NR1H4, ESR1, BBS5, PASD1, FHL2, HEY1, FLT3, MLXIPL, TP73 | 116 |
| Histone H3 methyltransferase activity | 16/2460 | 29/10811 | 0.00015281 | 0.02087801 | 0.01987948 | KMT2D, NSD1, KMT2A, SETD1B, ASH1L, SETD2, EZH2, KMT2C, PRDM2, SETD3, EHMT1, NSD3, SETDB1, MECOM, METTL23, SMYD3 | 16 |
| RNA polymerase II-specific DNA-binding transcription factor binding | 85/2460 | 262/10811 | 0.00017408 | 0.02087801 | 0.01987948 | SPEN, CTDP1, NSD1, NCOA1, NCOR2, CITED2, EP300, TACC1, ARID1A, NCOR1, BAZ2A, HIPK2, MED12, HMGB1, MED30, VDR, MED13, CHD4, MED4, CNOT1, SP1, ATF2, TAF1, ASXL1, TOB2, PPARG, LDB1, RBX1, HNF4A, SIN3A, THRAP3, NOTCH2, FOXP1, ZBTB7A, DDX5, COMMD6, MED25, PARK7, UBXN7, SRC, MTDH, PPARGC1B, MED1, ZFPM1, TAF11, SETD3, TP53BP1, HMGN3, TEAD3, NRIP1, ETS2, PSMD10, MDFIC, TRIM32, TCF3, TRERF1, GATA3, LATS1, STK4, EPAS1, KDM3A, MEF2D, TTC8, PTPN2, EGR2, TAF10, MAD2L2, ESRRB, PARP1, COMMD7, PARP9, SMARCE1, NR4A1, SPI1, PROX1, HMGA2, C1D, AR, NR1H4, ESR1, BBS5, HEY1, FLT3, MLXIPL, TP73 | 85 |
| Nuclear receptor binding | 42/2460 | 110/10811 | 0.00018176 | 0.02087801 | 0.01987948 | NSD1, NCOA1, NCOR2, EP300, TACC1, ARID1A, NCOR1, BAZ2A, MED12, MED30, VDR, MED13, MED4, CNOT1, TAF1, ASXL1, TOB2, PPARG, THRAP3, FOXP1, ZBTB7A, DDX5, MED25, PARK7, SRC, PPARGC1B, MED1, TAF11, HMGN3, NRIP1, TRERF1, LATS1, KDM3A, TAF10, PARP1, SMARCE1, NR4A1, PROX1, C1D, NR1H4, ESR1, FLT3 | 42 |
| Transcription corepressor activity | 49/2460 | 134/10811 | 0.00019108 | 0.02087801 | 0.01987948 | SPEN, NSD1, PFDN5, NCOR2, CITED2, ATF7IP, YAF2, RERE, NCOR1, HIPK2, HMGB1, SUFU, RBFOX2, KDM5B, ATN1, EZH2, TOB2, TFAP2A, TLE3, PHF12, SIN3A, URI1, HSBP1, BCORL1, CCND1, NCOA5, KMT5A, MED1, ZFPM1, PIAS1, NRIP1, TCF25, SRSF2, TRERF1, GMNN, MYBBP1A, TBL1X, SFMBT2, PARP9, NOC2L, HMGA2, C1D, PIAS4, MTA3, MIER3, CBFA2T3, FHL2, MIER2, DNMT3B | 49 |
| Structural molecule activity | 137/2460 | 459/10811 | 0.00019492 | 0.02087801 | 0.01987948 | SPTBN2, RPL11, AGRN, RPL10A, RPS15, LAMA5, RPS27L, RPS3A, RPL18A, MAP1A, MACF1, RPL32, RPL22L1, RPL15, RPL26L1, RPS14, RPS21, NUP98, NUMA1, RPL31, POM121, RPS12, RPL35A, MRPS24, DAP3, MRPS6, ERC1, RPL19, COPA, PRELP, CMTM8, LAD1, EFEMP1, EIF3A, DCN, RPL3, CLDN10, IGFBP7, NUP214, RPSA, ARPC3, RPS20, SPTBN1, STX2, MRPL54, RPL8, VILL, RPS16, MRPS18A, LMNA, RPLP1, EPB42, RPL13A, UBA52, RPS19, CRYBG3, EVPL, LUM, MRPS35, MRPL32, MRPL41, MRPL43, RPL37, NEXN, TPR, HSPG2, MRPL36, SPTAN1, FBLN2, DAG1, VPS25, CLDN1, RPL24, MRPL15, VWA1, LLGL1, JUP, PLS1, PCLO, DCTN3, CTHRC1, OGN, RPL9, RPLP0, TFPI2, COL4A1, TGFBI, FBN1, MRPS33, SEC31B, YEATS4, MRPL37, MRPL46, SEC13, MRPL51, HOMER1, BSN, RPS7, CSRP2, CCDC6, MFAP5, RSL24D1, NID1, CROCC, COL27A1, RPL34, OBSCN, COL5A2, EPB41, LMNB2, MRPS31, TUBGCP4, THBS2, MRPS14, PPL, MRPS18C, RPL14, NUP54, CLDN5, HAPLN4, NPHP4, COL7A1, ANK1, SNTB2, COL6A3, TUBE1, MATN2, COL11A1, LAMB3, ANK2, SRPX2, COL28A1, TUBG1, EDIL3, COL3A1, MXRA5, HMCN2 | 137 |
