# Supplementary material for: Tissue tropism, pathology, and pathogenesis of West Nile virus infection in saltwater crocodile (Crocodylus porosus)
Source: PLoS Negl Trop Dis. 2025 Aug 4;19(8):e0013385. doi: 10.1371/journal.pntd.0013385 (PMC12331170; doi:10.1371/journal.pntd.0013385)
Supplement: S5 Table — (DOCX) [file pntd.0013385.s005.docx]

**S5 Table.** Genes expressed in kidney during early response to infection

| **Gene cluster** | **Gene** | **Name** | **fold change (log_2_ transformed)** | **Adjusted p value** |
| --- | --- | --- | --- | --- |
| Cluster 1 | IRX1 | Iroquois Homeobox 1 | -1.221493 | 0.040258006 |
|  | PNP | Purine Nucleoside Phosphorylase | -1.029572 | 0.022939499 |
|  | RNF223 | Ring Finger Protein 223 | -2.111767 | 0.014266596 |
|  | MAT1A | Methionine Adenosyltransferase 1A | -1.747216 | 3.60E-05 |
|  | SLC25A34 | Solute Carrier Family 25 Member 34 | -1.147084 | 8.94E-05 |
|  | TMEM238 | Transmembrane Protein 238 | -1.392816 | 0.039688288 |
|  | SLC26A1 | Solute Carrier Family 26 Member 1 | -0.832775 | 0.003811282 |
|  | KLF15 | KLF Transcription Factor 15 | -1.355110 | 0.00023626 |
|  | FZD5 | Frizzled Class Receptor 5 | -1.190005 | 0.00520643 |
|  | BCAT2 | Branched Chain Amino Acid Transaminase 2 | -1.062097 | 0.001734498 |
|  | RASSF7 | Ras Association Domain Family Member 7 | -0.847397 | 0.001734498 |
| Cluster 2 | LOC109316215 (TRIM27L) | Zinc finger protein RFP-like | 2.356125 | 0.005192261 |
|  | LOC109314980 (SAMD9L) | Sterile Alpha Motif Domain Containing 9 Like | 1.045643 | 0.027196436 |
|  | LOC109309990 (TRIM25L) | E3 ubiquitin/ISG15 ligase TRIM25-like | 1.191257 | 0.039753507 |
|  | LOC109318466 (IFI27L2) | Interferon Alpha Inducible Protein 27-like protein 2A | 2.389631 | 9.25E-05 |
|  | LOC109321967 (HERC5) | E3 ISG15-protein ligase HERC5-like | 2.152325 | 0.004158698 |
|  | LOC109324007 (IFIT5) | Interferon Induced Protein With Tetratricopeptide Repeats 5 | 3.626428 | 0.00049915 |
|  | USP18 | Ubiquitin Specific Peptidase 18 | 3.017020 | 0.00279001 |
|  | LOC109313318 (IFI27L2a) | Interferon alpha-inducible protein 27, mitochondrial-like | 4.165634 | 4.42E-06 |
|  | LOC109309480 (CXCL10-like) | C-X-C motif chemokine 10-like | 2.072022 | 0.040169484 |
| Cluster 3 | MUC2 | Mucin 2, Oligomeric Mucus/Gel-Forming | 6.629645 | 0.003591461 |
|  | MMP7 | Matrix metalloproteinase 7 | 4.070412 | 0.043387436 |
|  | RPS21 | Ribosomal Protein S21 | 1.469663 | 0.000617703 |
|  | SRGN | Serglycin | 1.452780 | 0.02356484 |
|  | DSCC1 | DNA Replication and Sister Chromatid Cohesion 1 | 1.442386 | 0.02717644 |
|  | DISC1 | DISC1 Scaffold Protein | 1.439305 | 0.043179146 |
|  | CARS2 | Cysteinyl-TRNA Synthetase 2, Mitochondrial | 1.118564 | 0.001734498 |
|  | DNAJC15 | DnaJ Heat Shock Protein Family (Hsp40) Member C15 | 1.106237 | 0.026562133 |
|  | DNAJC1 | DnaJ Heat Shock Protein Family (Hsp40) Member C15 | 1.079301 | 0.010907677 |
|  | PCNP | PEST Proteolytic Signal Containing Nuclear Protein | 1.075757 | 0.004158698 |
|  | HPGD | 15-Hydroxyprostaglandin Dehydrogenase | 1.072842 | 0.004158698 |
|  | CPNE8 | Copine 8 | 1.068167 | 0.005713289 |
|  | CCDC91 | Coiled-Coil Domain Containing 91 | 1.050821 | 0.019803696 |
| Cluster 4 | JTB | Jumping Translocation Breakpoint | 1.115847 | 0.00708294 |
|  | NREP | Neuronal Regeneration Related Protein | 1.039546 | 0.009150035 |
|  | NEXN | Nexilin F-Actin Binding Protein | 1.369449 | 0.022651405 |
|  | EPHX4 | Epoxide Hydrolase 4 | 1.252278 | 0.034814982 |
|  | FABP3 | Fatty Acid Binding Protein 3 | 1.410042 | 3.76E-05 |
