# Supplementary material for: Tissue tropism, pathology, and pathogenesis of West Nile virus infection in saltwater crocodile (Crocodylus porosus)
Source: PLoS Negl Trop Dis. 2025 Aug 4;19(8):e0013385. doi: 10.1371/journal.pntd.0013385 (PMC12331170; doi:10.1371/journal.pntd.0013385)
Supplement: S6 Table — (DOCX) [file pntd.0013385.s006.docx]

**S6 Table.** Genes expressed in kidney during late response to infection

| **Gene cluster** | **Gene** | **Name** | **Fold change (log_2_ transformed)** | **Adjusted p value** |
| --- | --- | --- | --- | --- |
| Cluster 1 | SPTLC2 | Serine palmitoyltransferase, long chain base subunit 2 | 3.365054 | 5.04E-22 |
|  | RPS3A | Ribosomal protein S3 | 2.449695 | 3.23E-28 |
|  | CARS2 | Cysteinyl-TRNA Synthetase 2, Mitochondrial | 3.032636 | 4.16E-47 |
|  | NIPSNAP3A | Nipsnap Homolog 3A | 1.964285 | 1.07E-22 |
|  | LOC109309857 | Follistatin-related protein 4 | 2.555573 | 1.26E-21 |
|  | CARS2 | Cysteinyl-TRNA Synthetase 2, Mitochondrial | 3.032636 | 4.16E-47 |
|  | RPL10A | Ribosomal Protein L10a | 2.197646 | 2.68E-26 |
|  | RPL11 | Ribosomal Protein L11 | 2.935072 | 1.03E-46 |
|  | LAMTOR5 | Ragulator complex protein LAMTOR5 | 1.873978 | 1.84509E-19 |
|  | MSRB2 | Methionine Sulfoxide Reductase B2 | 2.298814 | 5.25E-29 |
|  | LOC109315013 (NME1) | Nucleoside diphosphate kinase A | 2.004959 | 5.02E-22 |
| Cluster 2 | SLC41A2 | Solute Carrier Family 41 Member 2 | 2.119394 | 2.84E-20 |
|  | RPS15 | Ribosomal Protein S15 | 2.145310 | 5.26E-26 |
|  | RPL18A | Large ribosomal subunit protein eL18A | 1.977829 | 1.48E-19 |
|  | NDUFS4 | NADH:Ubiquinone Oxidoreductase Subunit S4 | 2.298660 | 9.14E-25 |
|  | NFYB | Nuclear Transcription Factor Y Subunit Beta | 2.007063 | 3.17E-25 |
|  | CUNH18orf32 | R032 protein | 2.355365 | 2.92E-24 |
|  | ISOC1 | Isochorismatase domain-containing protein 1 | 2.365686 | 3.85E-28 |
|  | CDC14A | Cell Division Cycle 14A | 2.105276 | 1.19E-22 |
|  | RPS27L | Small Ribosomal Protein S27 eS27-like | 1.936789 | 3.17E-22 |
|  | GAPDH | Glyceraldehyde-3-phosphate dehydrogenase | 2.118740 | 6.46E-28 |
| Cluster 3 | SRGN | Serglycin | 1.452780 | 0.02356484 |
|  | DBI | Diazepam Binding Inhibitor aka Acyl-CoA Binding Protein | 1.497254 | 0.000103624 |
|  | PAIP2 | Polyadenylate-binding protein-interacting protein 2 | 2.247270 | 5.33E-22 |
|  | TBCA | Tubulin Folding Cofactor A | 2.364786 | 9.72E-23 |
|  | DNAJC1 | DnaJ Heat Shock Protein Family (Hsp40) Member C15 | 2.384202 | 1.58E-23 |
| Cluster 4 | TMSB15B | Thymosin Beta 15B | 2.438136 | 7.43E-31 |
|  | UBL5 | Ubiquitin Like 5 | 2.316016 | 1.75E-19 |
|  | UBE2W | Ubiquitin Conjugating Enzyme E2 W | 2.534430 | 5.90E-22 |
|  | GPX4 | Glutathione peroxidase 4 | 2.199220 | 1.65E-24 |
|  | CTDP1 | CTD Phosphatase Subunit 1 | 2.565856 | 3.55E-33 |
|  | CD3D | T-cell surface glycoprotein CD3 delta chain | 2.051193 | 1.47E-19 |
|  | DAP | Death Associated Protein | 2.609390 | 5.59E-27 |
|  | MPHOSPH6 | M-Phase Phosphoprotein 6 | 2.497392 | 3.32E-19 |
|  | PLAC9 | Placenta-specific 9 | 2.203028 | 4.52E-20 |
|  | POMP | Proteasome Maturation Protein | 2.662003 | 1.86E-24 |
|  | CCDC91 | Coiled-Coil Domain Containing 91 | 2.762841 | 1.28E-27 |
|  | DISC1 | DISC1 Scaffold Protein | 3.028014 | 5.79E-20 |
|  | YAF2 | YY1-associated factor 2 | 2.365306 | 1.07E-19 |
|  | KIAA1107 (currently known as BTBD8) | BTB Domain Containing 8 | 3.260904 | 6.62E-40 |
|  | PFDN5 | Prefoldin Subunit 5 | 2.511625 | 1.04E-25 |
|  | HPGD | 15-Hydroxyprostaglandin Dehydrogenase | 2.878541 | 2.89E-34 |
|  | LOC109317815 (RPS10) | Ribosomal protein S10 | 2.537037 | 9.80E-26 |
|  | NDUFA4 | NADH dehydrogenase 1 alpha subcomplex 4 | 2.118941 | 1.68E-19 |
|  | ARL6IP6 | ADP Ribosylation Factor Like GTPase 6 Interacting Protein 6 | 2.902931 | 2.77E-28 |
|  | FABP3 | Fatty Acid Binding Protein 3 | 3.345215 | 9.57E-33 |
| Cluster 5 | UCHL1 | Ubiquitin carboxyl-terminal hydrolase isozyme L1 | 2.157018 | 8.72E-21 |
|  | SUSD3 | Sushi Domain Containing 3 | 2.102597 | 1.68E-24 |
|  | RWDD4 | RWD domain-containing protein 4 | 2.275977 | 1.43E-23 |
|  | POLR1D | RNA polymerase I and III subunit D | 2.093569 | 7.10E-20 |
|  | LAMTOR2 | lysosomal adaptor and MAPK and mammalian target of Rapamycin (mTOR) activator (LAMTOR) | 2.267488 | 5.03E-22 |
|  | MICU3 | Mitochondrial Calcium Uptake Family Member 3 | 2.525100 | 1.32E-30 |
|  | JTB | Jumping Translocation Breakpoint | 2.914710 | 1.20E-30 |
|  | MPC1 | Mitochondrial pyruvate carrier 1 | 2.814837 | 3.63E-36 |
|  | NREP | Neuronal Regeneration Related Protein | 3.107706 | 2.28E-35 |
|  | CPNE8 | Copine-8 | 2.357559 | 4.17E-26 |
|  | TIMM8A | Translocase Of Inner Mitochondrial Membrane 8A | 2.356139 | 8.32E-25 |
|  | NME7 | Nucleoside diphosphate kinase homolog 7 | 2.361550 | 2.90E-19 |
|  | UBL3 | Ubiquitin Like 3 | 1.941725 | 9.27E-22 |
|  | DCAF5 | DDB1 and CUL4 associated factor 5 | 2.545480 | 7.47E-36 |
|  | AAED1 | AhpC/TSA Antioxidant Enzyme Domain-Containing Protein 1 | 2.050673 | 8.53E-20 |
|  | DNAJC19 | DnaJ Heat Shock Protein Family (Hsp40) Member C19 | 2.309124 | 6.02E-22 |
|  | MAGOH | Mago Homolog, Exon Junction Complex Subunit | 3.002860 | 9.01E-33 |
