# Supplementary material for: Tissue tropism, pathology, and pathogenesis of West Nile virus infection in saltwater crocodile (Crocodylus porosus)
Source: PLoS Negl Trop Dis. 2025 Aug 4;19(8):e0013385. doi: 10.1371/journal.pntd.0013385 (PMC12331170; doi:10.1371/journal.pntd.0013385)
Supplement: S7 Table — (DOCX) [file pntd.0013385.s007.docx]

**S7 Table.** Gene ontology categories in the liver during early – stage response infection

| **Description** | **GeneRatio** | **BgRatio** | **p value** | **p. adjust** | **q value** | **Gene ID** | **Count** |
| --- | --- | --- | --- | --- | --- | --- | --- |
| Negative regulation of lipase activity | 2/14 | 10/10210 | 7.81E-05 | 0.016020288 | 0.012848351 | ANGPTL3, ANGPTL4 | 2 |
| Regulation of lipoprotein lipase activity | 2/14 | 11/10210 | 9.54E-05 | 0.016020288 | 0.012848351 | ANGPTL3, ANGPTL4 | 2 |
| Acylglycerol homeostasis | 2/14 | 26/10210 | 0.000556899 | 0.046779511 | 0.037517402 | ANGPTL3, ANGPTL4 | 2 |
| Triglyceride homeostasis | 2/14 | 26/10210 | 0.000556899 | 0.046779511 | 0.037517402 | ANGPTL3, ANGPTL4 | 2 |
| External encapsulating structure | 4/15 | 369/10589 | 0.00145917 | 0.02991299 | 0.026879451 | ANGPTL3, ANGPTL4, ELFN2, COL5A3 | 4 |
| Extracellular matrix | 4/15 | 369/10589 | 0.00145917 | 0.02991299 | 0.026879451 | ANGPTL3, ANGPTL4, ELFN2, COL5A3 | 4 |
