# Supplementary material for: Tissue tropism, pathology, and pathogenesis of West Nile virus infection in saltwater crocodile (Crocodylus porosus)
Source: PLoS Negl Trop Dis. 2025 Aug 4;19(8):e0013385. doi: 10.1371/journal.pntd.0013385 (PMC12331170; doi:10.1371/journal.pntd.0013385)
Supplement: S8 Table — (DOCX) [file pntd.0013385.s008.docx]

**S8 Table.** Gene ontology categories in the liver during late – stage response infection

| **Description** | **Gene Ratio** | **Bg Ratio** | **p value** | **p. adjust** | **q value** | **geneID** | **Count** |
| --- | --- | --- | --- | --- | --- | --- | --- |
| Regulation of purine nucleotide metabolic process | 6/86 | 63/10257 | 1.35E-05 | 0.0162564 | 0.01489998 | MLXIPL, TMSB4X, NOS2, PDK4, GPD1, HPCA | 6 |
| Regulation of nucleotide metabolic process | 6/86 | 64/10257 | 1.48E-05 | 0.0162564 | 0.01489998 | MLXIPL, TMSB4X, NOS2, PDK4, GPD1, HPCA | 6 |
| Regulation of nucleotide biosynthetic process | 4/86 | 23/10257 | 3.61E-05 | 0.019839578 | 0.018184181 | TMSB4X, NOS2, PDK4, HPCA | 4 |
| Regulation of purine nucleotide biosynthetic process | 4/86 | 23/10257 | 3.61E-05 | 0.019839578 | 0.018184181 | TMSB4X, NOS2, PDK4, HPCA | 4 |
| Regulation of small molecule metabolic process | 9/86 | 223/10257 | 9.94E-05 | 0.032282872 | 0.029589217 | MLXIPL, APOA4, TMSB4X, PTH1R, ACACB, NOS2, PDK4, GPD1, HPCA | 9 |
| Positive regulation of nucleotide metabolic process | 4/86 | 30/10257 | 0.000106899 | 0.032282872 | 0.029589217 | MLXIPL, TMSB4X, NOS2, GPD1 | 4 |
| Positive regulation of purine nucleotide metabolic process | 4/86 | 30/10257 | 0.000106899 | 0.032282872 | 0.029589217 | MLXIPL, TMSB4X, NOS2, GPD1 | 4 |
| Response to wounding | 1286 | 400/10257 | 0.000117553 | 0.032282872 | 0.029589217 | TNC, GRIN2C, ACTA2, APOA4, NREP, JUN, GRHL3, B4GALT1, SYT7, TUBB1, HRG, NRG1 | 12 |
| Regeneration | 7/86 | 141/10257 | 0.000173689 | 0.042399422 | 0.038861651 | TNC, APOA4, NREP, JUN, FPGS, IGFBP1, FZD9 | 7 |
| Positive regulation of small molecule metabolic process | 6/86 | 102/10257 | 0.000206212 | 0.045304716 | 0.041524529 | MLXIPL, APOA4, TMSB4X, PTH1R, NOS2, GPD1 | 6 |
