# Supplementary material for: Tissue tropism, pathology, and pathogenesis of West Nile virus infection in saltwater crocodile (Crocodylus porosus)
Source: PLoS Negl Trop Dis. 2025 Aug 4;19(8):e0013385. doi: 10.1371/journal.pntd.0013385 (PMC12331170; doi:10.1371/journal.pntd.0013385)
Supplement: S9 Table — (DOCX) [file pntd.0013385.s009.docx]

**S9 Table.** Genes expressed in liver during early response to infection

| **Gene cluster** | **Gene** | **Name** | **Fold change (log_2_ transformed)** | **Adjusted p value** |
| --- | --- | --- | --- | --- |
| Cluster 1 | SLC30A2 | Solute Carrier Family 30 Member 2 | -1.625713996 | 0.002109441 |
|  | HSD17B7 | Hydroxysteroid 17-beta dehydrogenase 7 | -1.101646686 | 0.024176076 |
|  | ANGPTL3 | Angiopoietin-related protein 3 | -1.962326480 | 0.000000006 |
| Cluster 2 | TENM4 | Teneurin Transmembrane Protein 4 | 2.313256476 | 0.015784143 |
|  | LOC109318131 (FADS2) | Fatty Acid Desaturase 2 | 2.175448060 | 0.024176076 |
| Cluster 3 | GIPC2 | GIPC PDZ Domain Containing Family Member 2 | 2.608545797 | 0.028430117 |
|  | ANGPTL4 | Angiopoietin-related protein 4 | 2.558686858 | 0.014206760 |
|  | DHX58 | DExH-box helicase 58 | 2.449439699 | 0.048455845 |
|  | LOC109323658 (SLC27A6) | Solute carrier family 27-member 6 | 2.379103345 | 0.002271436 |
|  | TENM4 | Teneurin Transmembrane Protein 4 | 2.313256476 | 0.015784143 |
|  | LOC109318131 (FADS2) | Fatty Acid Desaturase 2 | 2.175448060 | 0.024176076 |
|  | ELFN2 | Protein phosphatase 1 regulatory subunit 29 | 2.061583861 | 0.023699904 |
|  | LOC109310705 (CIDEC) | Cell Death Inducing DFFA Like Effector C | 1.877722303 | 0.024176076 |
|  | GAMT | Guanidinoacetate N-Methyltransferase | 1.251616839 | 0.024176076 |
|  | TUB | Tubby protein | 1.095085715 | 0.049728649 |
|  | LOC109305775 (IL10RB) | Interleukin 10 Receptor Subunit Beta | -0.925148817 | 0.024176076 |
|  | DHX38 | DEAH-box helicase 38 | -0.949728162 | 0.007888972 |
