# Supplementary material for: Tissue tropism, pathology, and pathogenesis of West Nile virus infection in saltwater crocodile (Crocodylus porosus)
Source: PLoS Negl Trop Dis. 2025 Aug 4;19(8):e0013385. doi: 10.1371/journal.pntd.0013385 (PMC12331170; doi:10.1371/journal.pntd.0013385)
Supplement: S10 Table — (DOCX) [file pntd.0013385.s010.docx]

**S10 Table.** Genes expressed in liver during late response to infection

| **Gene cluster** | **Gene** | **Name** | **Fold change (log_2_ transformed)** | **Adjusted p value** |
| --- | --- | --- | --- | --- |
| Cluster 1 and 2 | LOC109320129 (CA2-like) | Carbonic anhydrase 2-like | 1.377428412 | 0.013734968 |
|  | NREP | Neuronal Regeneration Related Protein | 1.335017268 | 0.006489487 |
|  | ANGPTL4 | Angiopoietin-like 4 | 1.945713789 | 0.034398977 |
|  | CRAT | Carnitine O-acetyltransferase | 1.168924165 | 0.014087466 |
|  | FZD9 | Frizzled Class Receptor 9 | 1.309408007 | 0.046310412 |
|  | RERG | RAS Like Estrogen Regulated Growth Inhibitor | 1.701102702 | 0.002493553 |
|  | LOC109323658 (SLC27A6) | Solute carrier family 27-member 6 | 2.379103345 | 0.002271436 |
|  | LOC109322021 (FXI) | Coagulation Factor XI-like | 1.000897007 | 0.025022944 |
|  | LOC109308482 (FETUB) | Fetuin-B-like | 1.165112 | 2.45E-02 |
|  | LOC109321718 (SERPINA1-like) | Alpha-1 antitrypsin (AAT) | 1.625823724 | 0.016980592 |
|  | HRG | Histidine Rich Glycoprotein | 1.185832615 | 0.0254202 |
|  | UCHL1 | Ubiquitin carboxyl-terminal hydrolase isozyme L1 | 1.507674821 | 0.008485791 |
|  | LOC109305881 (CD276) | CD276 antigen homolog | 1.049072269 | 0.022224468 |
|  | EPB42 | Erythrocyte Membrane Protein Band 4.2 | 4.283825744 | 0.006586801 |
|  | NKAIN2 | Sodium/Potassium Transporting ATPase Interacting 2 | 1.253944178 | 0.001374171 |
|  | KCNQ1 | Potassium Voltage-Gated Channel Subfamily Q Member 1 | 2.186590149 | 0.030927204 |
|  | XIRP1 | Xin actin-binding repeat-containing protein 1 | 1.872077632 | 0.013640792 |
|  | RASSF9 | Ras Association Domain Family Member 9 | 1.479265125 | 0.011297558 |
|  | LOC109316307 (CDKN1A) | Cyclin Dependent Kinase Inhibitor 1 | 1.318815299 | 0.003636778 |
|  | LOC109318169 (DSA2B) | Dispanin subfamily A member 2b | 1.115834826 | 0.049349803 |
| Cluster 3 | LOC109310705 (CIDE-3) | Cell death-inducing DFFA-like effector C (CIDEC) | 1.823216304 | 0.010765983 |
|  | GPD1 | Glycerol-3-Phosphate Dehydrogenase 1 | 1.490301311 | 0.040483045 |
|  | THRSP | Thyroid Hormone Responsive | 1.076175443 | 0.039447141 |
|  | LOC109324215 (TMEM150A) | Transmembrane protein 150A (damage-regulated autophagy modulator 5) | 1.865054068 | 0.004068811 |
|  | B4GALT1 | Beta-1,4-galactosyltransferase 1 | 1.031480847 | 0.015690946 |
|  | LOC109322480 (LY6E-like) | Lymphocyte antigen 6 family member E-like | 1.405591121 | 0.022971558 |
|  | NOS2 | Nitric Oxide Synthase 2 | 3.147859951 | 0.017520953 |
|  | LOC109318466 | Interferon alpha-inducible protein 27-like protein 2A | 4.285098909 | 0.012166722 |
|  | RSAD2 (previously known as Viperin) | Radical S-adenosyl methionine domain-containing protein 2 | 4.618054577 | 0.010765983 |
|  | DHX58 | DExH-box helicase 58 | 2.247251468 | 0.012166722 |
|  | PLAC9 | Placenta Associated 9 | 2.472368617 | 0.01098887 |
|  | LOC109305835 (MX1) | MX dynamin-like GTPase 1 | 2.947659400 | 0.005874655 |
|  | GRHL3 | Grainyhead-like 3 transcription factor | 2.026320414 | 0.010472555 |
|  | LOC109318168 (IFITM-like) | Interferon-induced transmembrane protein 1-like | 1.481846402 | 0.015690946 |
|  | ACTA2 | Actin Alpha 2, Smooth Muscle | 1.421197493 | 0.000316994 |
|  | GRIP2 | Glutamate receptor-interacting protein 2 | 1.319723008 | 0.025022944 |
|  | LOC109308130 (ERAP2) | Endoplasmic reticulum aminopeptidase 2 | 2.280944072 | 0.030095047 |
|  | LEAP2 | Liver enriched antimicrobial peptide 2 | 1.137419818 | 0.031857721 |
|  | GIPC2 | GIPC PDZ Domain Containing Family Member 2 | 2.777684772 | 0.001171259 |
|  | WFIKKN1 | Growth and differentiation factor Associated Serum Protein 1 | 7.775361462 | 3.86E-05 |
|  | AFF3 | ALF transcription elongation factor 3 | 1.510366237 | 0.021277919 |
|  | LGALS9 | Galectin 9 | 2.816747107 | 0.035270386 |
| Cluster 4 | CRLF2 | Cytokine receptor-like factor 2 | 1.036758268 | 0.001684911 |
|  | APOA4 | Apolipoprotein A4 | 1.099266372 | 0.002039159 |
|  | GATM | Glycine amidinotransferase, mitochondrial | 1.872298545 | 0.000797413 |
|  | LOC109315166 (CYP2C5) | P450 2C5-like | 1.074726985 | 0.001041975 |
|  | PRR29 | Proline-rich protein 29 | 1.017203168 | 0.003350614 |
|  | CACNA1D | Calcium Voltage-Gated Channel Subunit Alpha1 D | 1.375724878 | 0.010765983 |
|  | LOC109318131 (FADS2L) | Fatty acid desaturase 2-like | 2.696876290 | 0.025022944 |
|  | SLC26A5 | Solute Carrier Family 26 Member 5 (Prestin) | 1.412162988 | 1.30E-05 |
|  | HTR2C | 5-Hydroxytryptamine Receptor 2C | 1.723817057 | 0.04807904 |
|  | LOC109308457 (MRP1-like) | Multidrug Resistance Protein 1 | 1.565738634 | 6.46E-06 |
|  | SLC16A1 | Solute Carrier Family 16 Member 1 | 1.371615959 | 1.62E-07 |
|  | ENTPD5 | Ectonucleoside triphosphate diphosphohydrolase 5 | 1.036552927 | 2.41E-05 |
|  | IL15 | Interleukin-15 | 1.039000559 | 0.01098887 |
| Cluster 5 | GHR | Growth Hormone Receptor | 1.310464532 | 0.013174619 |
|  | TMEM254 | Transmembrane Protein 254 | 1.702007695 | 0.017520953 |
|  | NDRG1 | N-Myc Downstream Regulated 1 | 1.285970062 | 0.003752796 |
|  | PARP9 | Poly(ADP-Ribose) Polymerase Family Member 9 | 1.218380421 | 0.000408382 |
|  | LOC109315331 (Ovotransferrin (conalbumin) | Ovotransferrin | 1.116214183 | 0.002101144 |
|  | APELA | Apelin Receptor Early Endogenous Ligand | 1.783830372 | 0.000408382 |
|  | SLCO1A2 | Solute carrier organic anion transporter family member 1A2 | 1.639280609 | 0.034831266 |
|  | LOC109311477 (CYP39A1-like) | Cytochrome P450 Family 39 Subfamily A Member 1 | 1.582163578 | 0.002537207 |
|  | HHIP | Hedgehog Interacting Protein | 1.704925586 | 0.01098887 |
|  | LOC109315145 (RDH2L) | Epidermal retinol dehydrogenase 2-like | 1.865109164 | 0.006087483 |
|  | LOC109318467 (IFI27L2A) | Interferon alpha-inducible protein 27-like protein 2A | 3.379345665 | 1.72E-16 |
|  | TMSB4X | Thymosin beta 4 | 1.027995402 | 0.002783453 |
| Cluster 6 | SPIDR | Scaffold Protein Involved in DNA Repair | -1.467049527 | 0.010765983 |
|  | QRICH2 | Glutamine Rich 2 | -1.823716857 | 0.017228165 |
|  | PTH1R | Parathyroid hormone/parathyroid hormone-related peptide receptor | -1.435641834 | 0.009731476 |
|  | CERCAM | Cerebral endothelial cell adhesion molecule | -1.453033741 | 0.025022944 |
|  | LOC109317005 (ARHGAP26) | Rho GTPase Activating Protein 4 | -3.265542581 | 0.042689222 |
|  | DNAJC12 | DnaJ heat shock protein family (Hsp40) member C12 | -3.222954665 | 0.040333177 |
|  | FAXDC2 | Fatty Acid Hydroxylase Domain Containing 2 | -3.734942702 | 0.026248689 |
|  | SYT7 | Synaptotagmin 7 | -1.543737467 | 0.015695422 |
|  | LOC109307902 (MOCS2) | Molybdenum Cofactor Synthesis 2 | -1.571264842 | 0.013640792 |
|  | FAM134B aka RETREG1 | Reticulophagy regulator 1 | -1.535123579 | 0.013734968 |
|  | LRRC66 | Leucine Rich Repeat Containing 66 | -1.999957845 | 0.001377906 |
|  | ATOH7 | Atonal BHLH Transcription Factor 7 | -1.519232667 | 0.045227233 |
|  | IGFBP1 | Insulin Like Growth Factor Binding Protein 1 | -1.537690516 | 0.020694693 |
|  | PDK4 | [Pyruvate dehydrogenase (acetyl-transferring)] kinase isozyme 4, mitochondrial | -1.622787084 | 0.018319929 |
|  | SEL1L3 | SEL1L Family Member 3 | -2.166514908 | 0.0254202 |
|  | GTPBP4 | GTP Binding Protein 4 | -1.011490539 | 0.013734968 |
|  | FRMD4B | FERM Domain Containing 4B | -1.257551101 | 0.019687939 |
