# Supplementary material for: Tissue tropism, pathology, and pathogenesis of West Nile virus infection in saltwater crocodile (Crocodylus porosus)
Source: PLoS Negl Trop Dis. 2025 Aug 4;19(8):e0013385. doi: 10.1371/journal.pntd.0013385 (PMC12331170; doi:10.1371/journal.pntd.0013385)
Supplement: S11 Table — (DOCX) [file pntd.0013385.s011.docx]

**S11 Table.** Summary of p-values from Tukey's multiple comparisons test of the level of upregulated liver cytokine and transcription factor expression between the different timepoints^£^

| **Timepoint** | **Antiviral genes** | | | | **Cell proliferation and apoptosis gene** | | **Pro and anti-inflammatory genes** | | | | |
| --- | --- | --- | --- | --- | --- | --- | --- | --- | --- | --- | --- |
|  | **IRF3** | **OASL** | **Mx1-like** | **TFEB** | **Ki67** | **CASP9** | **IL-1 β like** | **IL-34** | **HIF-1-α** | **CSF1[M-CSF]** | **TGF Beta** |
| 1dpi vs. 2dpi | *p=*0.0920 | *p=*0.8516 | *p=*0.4718 | *p=*0.3006 | *p=*0.9442 | *p=*0.8011 | *p=*0.8413 | *p=*0.1776 | *p=*0.5096 | *p=*0.9997 | *p=*0.7020 |
| 1dpi vs. 3dpi | ****p=*0.0418** | *p=*0.1428 | *p=*0.7948 | *p=*0.2281 | *p=*0.1331 | *p=*0.0590 | *p=*0.1908 | *p=*0.1443 | *p=*0.8872 | *p*>0.9999 | *p=*0.2871 |
| 1dpi vs. 4dpi | *p=*0.9338 | *p=*0.9146 | ****p=*0.0211** | *p=*0.9504 | *p=*0.9122 | *p=*0.9441 | *p=*0.9343 | *p=*0.9477 | *p=*0.5179 | *p=*0.9935 | *p=*0.9383 |
| 1dpi vs. 5dpi | *p=*0.4378 | *p=*0.3675 | *p*>0.9999 | *p=*0.8097 | *p=*0.7180 | *p=*0.5934 | *p=*0.6914 | *p=*0.6776 | *p=*0.0917 | *p=*0.7119 | *p=*0.7883 |
| 1dpi vs. 6dpi | *p=*0.2920 | *p=*0.0985 | *p=*0.1346 | *p=*0.6000 | *p=*0.4069 | *p=*0.3924 | *p=*0.4614 | *p=*0.2610 | *p=*0.7513 | *p=*0.4133 | *p=*0.4784 |
| 1dpi vs. 7dpi | ****p=*0.0222** | ****p=*0.0212** | *p=*0.1880 | ****p=*0.0211** | - | - | - | - | - | - | - |
| 1dpi vs. 9dpi | ****p=*0.0327** | ****p=*0.0276** | *p=*0.2580 | ****p=*0.0211** | - | - | - | - | - | - | - |
| 2dpi vs. 3dpi | *p=*0.4285 | *p=*0.4635 | *p=*0.6464 | *p=*0.3884 | *p=*0.3944 | *p=*0.3777 | *p=*0.4395 | *p=*0.3820 | *p=*0.9641 | *p=*0.9936 | *p=*0.4253 |
| 2dpi vs. 4dpi | *p=*0.9975 | *p=*0.9610 | *p=*0.6037 | *p=*0.9610 | *p=*0.9520 | *p=*0.9799 | *p=*0.9555 | *p=*0.9936 | *p=*0.5050 | *p=*0.9998 | *p=*0.9539 |
| 2dpi vs. 5dpi | *p=*0.7403 | *p=*0.3693 | *p=*0.9979 | *p=*0.8548 | *p=*0.7412 | *p=*0.6771 | *p=*0.7462 | *p=*0.7630 | *p=*0.1385 | *p=*0.6436 | *p=*0.8024 |
| 2dpi vs. 6dpi | *p=*0.4132 | *p=*0.2349 | *p*>0.9999 | *p=*0.6193 | *p=*0.5629 | *p=*0.5520 | *p=*0.5342 | *p=*0.5080 | *p=*0.7788 | *p=*0.9356 | *p=*0.5594 |
| 2dpi vs. 7dpi | *p=*0.1156 | *p=*0.1359 | *p=*0.5094 | ****p=*0.0214** | - | - | - | - | - | - | - |
| 2dpi vs. 9dpi | *p=*0.1672 | *p=*0.1919 | *p=*0.8506 | ****p=*0.0448** | - | - | - | - | - | - | - |
| 3dpi vs. 4dpi | *p*>0.9999 | *p*>0.9999 | *p=*0.4932 | *p=*0.9971 | *p=*0.9996 | *p*>0.9999 | *p=*0.9992 | *p*>0.9999 | *p=*0.7147 | *p=*0.9984 | *p=*0.9949 |
| 3dpi vs. 5dpi | *p=*0.9333 | *p=*0.4555 | *p=*0.0925 | *p=*0.9387 | *p=*0.9330 | *p=*0.9166 | *p=*0.8668 | *p=*0.9071 | *p=*0.4256 | *p=*0.1241 | *p=*0.8935 |
| 3dpi vs. 6dpi | *p=*0.9656 | *p=*0.6870 | *p=*0.7086 | *p=*0.9564 | *p=*0.9878 | *p=*0.9860 | *p=*0.9888 | *p=*0.9603 | *p*>0.9999 | *p=*0.8776 | *p=*0.9635 |
| 3dpi vs. 7dpi | *p=*0.3293 | *p=*0.3497 | *p=*0.5265 | *p=*0.1892 | *p=*0.2475 | *p=*0.2639 | *p=*0.2445 | *p=*0.2691 | *p=*0.3639 | *p=*0.4799 | *p=*0.1860 |
| 3dpi vs. 9dpi | *p=*0.5640 | *p=*0.5867 | *p=*0.6127 | *p=*0.3413 | *p=*0.4056 | *p=*0.4328 | *p=*0.4737 | *p=*0.4631 | *p=*0.9966 | *p=*0.6468 | *p=*0.4185 |
| 3dpi vs. 11dpi | *p=*0.4670 | *p=*0.5152 | *p=*0.9664 | *p=*0.2781 | *p=*0.4335 | *p=*0.4549 | *p=*0.4292 | *p=*0.4395 | *p=*0.3933 | *p=*0.6368 | *p=*0.3657 |
| 3dpi vs. 15dpi | *p=*0.3231 | *p=*0.3943 | *p=*0.7959 | *p=*0.1730 | *p=*0.3107 | *p=*0.2949 | *p=*0.3097 | *p=*0.3242 | *p=*0.9183 | *p=*0.5569 | *p=*0.2484 |
| 3dpi vs. 21dpi | *p=*0.9995 | *p=*0.9530 | *p=*0.8242 | *p*>0.9999 | *p=*0.9994 | *p*>0.9999 | *p*>0.9999 | *p*>0.9999 | *p=*0.9036 | *p=*0.9872 | *p=*0.9804 |
| 4dpi vs. 5dpi | *p*>0.9999 | *p*>0.9999 | *p=*0.7817 | *p*>0.9999 | *p*>0.9999 | *p*>0.9999 | *p*>0.9999 | *p*>0.9999 | *p*>0.9999 | *p*>0.9999 | *p*>0.9999 |
| 4dpi vs. 6dpi | *p*>0.9999 | *p*>0.9999 | *p=*0.0901 | *p*>0.9999 | *p*>0.9999 | *p*>0.9999 | *p*>0.9999 | *p*>0.9999 | *p=*0.5009 | *p*>0.9999 | *p=*0.9997 |
| 5dpi vs. 6dpi | *p*>0.9999 | *p*>0.9985 | *p*>0.9927 | *p*>0.9999 | *p*>0.9999 | *p*>0.9999 | *p*>0.9999 | *p*>0.9999 | *p=*0.2788 | *p*>0.9999 | *p*>0.9999 |
| 7dpi vs. 9dpi | *******p*<0.0001** | *******p*<0.0001** | *******p*<0.0001** | *******p*<0.0001** | *******p*<0.0001** | *******p*<0.0001** | *******p*<0.0001** | *******p*<0.0001** | *******p*<0.0001** | *******p*<0.0001** | *******p*<0.0001** |
| 7dpi vs. 11dpi | - | - | - | - | *******p*<0.0001** | *******p*<0.0001** | *******p*<0.0001** | *******p*<0.0001** | *******p*<0.0001** | *******p*<0.0001** | *******p*<0.0001** |
| 7dpi vs. 15dpi | - | - | - | - | *******p*<0.0001** | *******p*<0.0001** | *******p*<0.0001** | *******p*<0.0001** | *******p*<0.0001** | *******p*<0.0001** | *******p*<0.0001** |
| 7dpi vs. 21dpi | *p*>0.9999 | *p*>0.9999 | *p*>0.9999 | *p*>0.9999 | *******p*<0.0001** | *******p*<0.0001** | *******p*<0.0001** | *******p*<0.0001** | *******p*<0.0001** | *******p*<0.0001** | *******p*<0.0001** |
| 9dpi vs. 11dpi | - | - | - | - | *******p*<0.0001** | *******p*<0.0001** | *******p*<0.0001** | *******p*<0.0001** | *******p*<0.0001** | *******p*<0.0001** | *******p*<0.0001** |
| 9dpi vs. 15dpi | - | - | - | - | *******p*<0.0001** | *******p*<0.0001** | *******p*<0.0001** | *******p*<0.0001** | *******p*<0.0001** | *******p*<0.0001** | *******p*<0.0001** |
| 9dpi vs. 21dpi | *p*>0.9999 | *p*>0.9999 | *p*>0.9999 | *p*>0.9999 | *******p*<0.0001** | *******p*<0.0001** | *******p*<0.0001** | *******p*<0.0001** | *******p*<0.0001** | *******p*<0.0001** | *******p*<0.0001** |
| 11dpi vs. 15dpi | *******p*<0.0001** | *******p*<0.0001** | *******p*<0.0001** | *******p*<0.0001** | *******p*<0.0001** | *******p*<0.0001** | *******p*<0.0001** | *******p*<0.0001** | *******p*<0.0001** | *******p*<0.0001** | *******p*<0.0001** |
| 11dpi vs. 21dpi | *p*>0.9999 | *p*>0.9999 | *p*>0.9999 | *p*>0.9999 | *******p*<0.0001** | *******p*<0.0001** | *******p*<0.0001** | *******p*<0.0001** | *******p*<0.0001** | *******p*<0.0001** | *******p*<0.0001** |
| 15dpi vs. 21dpi | *p*>0.9999 | *p*>0.9999 | *p*>0.9999 | *p*>0.9999 | *******p*<0.0001** | *******p*<0.0001** | *******p*<0.0001** | *******p*<0.0001** | *******p*<0.0001** | *******p*<0.0001** | *******p*<0.0001** |

^£^Tukey's multiple comparisons test was performed to compare means of a given antiviral cytokine and transcription factor gene expression at two timepoints in a given tissue (Figure 4 – 6). Significant statistical difference thresholds are **p* ≤ 0.05, ***p* ≤ 0.01, ****p* ≤ 0.001, *****p* ≤ 0.0001, ns = not significant (unmarked).
