# Supplementary material for: Tissue tropism, pathology, and pathogenesis of West Nile virus infection in saltwater crocodile (Crocodylus porosus)
Source: PLoS Negl Trop Dis. 2025 Aug 4;19(8):e0013385. doi: 10.1371/journal.pntd.0013385 (PMC12331170; doi:10.1371/journal.pntd.0013385)
Supplement: S12 Table — (DOCX) [file pntd.0013385.s012.docx]

**S12 Table .** Summary of p-values from Tukey's multiple comparisons test of the level of upregulated splenic cytokine and transcription factor expression between the different timepoints^£^

| **Timepoint** | **Antiviral genes** | | | | **Cell proliferation and apoptosis gene** | | **Pro and anti-inflammatory genes** | | | | |
| --- | --- | --- | --- | --- | --- | --- | --- | --- | --- | --- | --- |
|  | **IRF3** | **OASL** | **Mx1-like** | **TFEB** | **Ki67** | **CASP9** | **IL-1 β like** | **IL-34** | **HIF-1-α** | **CSF1[M-CSF]** | **TGF Beta** |
| 1dpi vs. 2dpi | *p=*0.8749 | *p=*0.2073 | *p=*0.7899 | *p=*0.7791 | *p=*0.9997 | *p=*0.7611 | *p=*0.3754 | *p=*0.2640 | *p=*0.9313 | *p=*0.9995 | *p=*0.9175 |
| 1dpi vs. 3dpi | *p*>0.9999 | *p*>0.9999 | *p=*0.0482 | *p=*0.9512 | *p=*0.9978 | *p=*0.9721 | *p=*0.9833 | *p*>0.9999 | *p=*0.8190 | *p=*0.8865 | *p=*0.9994 |
| 1dpi vs. 4dpi | *p=*0.9596 | *p=*0.9613 | *p=*0.9127 | *p=*0.8358 | *p=*0.9803 | *p=*0.8950 | *p=*0.7732 | *p=*0.9052 | *p=*0.9948 | *p=*0.9973 | *p=*0.9520 |
| 1dpi vs. 5dpi | *p=*0.9886 | *p=*0.9969 | *p=*0.2862 | *p=*0.8456 | *p=*0.3045 | *p=*0.8694 | *p=*0.9940 | *p*>0.9999 | *p*>0.9999 | *p=*0.9932 | *p*>0.9999 |
| 1dpi vs. 6dpi | *p=*0.9781 | *p=*0.9926 | *p=*0.6441 | *p=*0.9382 | *p=*0.9942 | *p=*0.9538 | *p=*0.9268 | *p=*0.9539 | *p=*0.8936 | *p=*0.9942 | *p=*0.9692 |
| 1dpi vs. 7dpi | - | - | - | - | - | - | *p=*0.9217 | *p=*0.9940 |  | *p=*0.9834 | *p=*0.9475 |
| 1dpi vs. 9dpi | - | - | - | - | *p=*0.8563 | *p=*0.8034 | *p=*0.6859 | *p=*0.8034 | *p=*0.9996 | *p=*0.9988 | *p=*0.7788 |
| 1dpi vs. 15dpi | *p=*0.9472 | *p=*0.9891 | *p=*0.8714 | *p=*0.4352 | - | - | - | - | - | - | - |
| 1dpi vs. 21dpi | *p=*0.9872 | *p=*0.9847 | *p=*0.4512 | *p=*0.6356 | - | - | - | - | - | - | - |
| 2dpi vs. 3dpi | *p*>0.9999 | *p*>0.9999 | *p=*0.6397 | *p*>0.9999 | *p*>0.9999 | *p*>0.9999 | *p*>0.9999 | *p*>0.9999 | *p*>0.9999 | *p*>0.9999 | *p*>0.9999 |
| 2dpi vs. 4dpi | *p=*0.9681 | *p=*0.9929 | *p=*0.9936 | *p=*0.9129 | *p=*0.9752 | *p=*0.9383 | *p=*0.8898 | *p=*0.9682 | *p*>0.9999 | *p*>0.9999 | *p=*0.9869 |
| 2dpi vs. 5dpi | *p=*0.9981 | *p*>0.9999 | *p=*0.3377 | *p=*0.9070 | *p=*0.2500 | *p=*0.9047 | *p*>0.9999 | *p*>0.9999 | *p*>0.9999 | *p*>0.9999 | *p*>0.9999 |
| 2dpi vs. 6dpi | *p=*0.9916 | *p*>0.9999 | *p=*0.7507 | *p=*0.9857 | *p=*0.9946 | *p=*0.9813 | *p=*0.9890 | *p=*0.9919 | *p=*0.9973 | *p*>0.9999 | *p=*0.9953 |
| 2dpi vs. 7dpi | - | - | - | - | - | - | *p=*0.9961 | *p*>0.9999 |  | *p*>0.9999 | *p=*0.9987 |
| 2dpi vs. 9dpi | - | - | - | - | *p=*0.9307 | *p=*0.9114 | *p=*0.8511 | *p=*0.9137 | *p=*0.9923 | *p*>0.9999 | *p=*0.9356 |
| 2dpi vs. 15dpi | *p=*0.9337 | *p*>0.9999 | *p*>0.9999 | *p=*0.5584 | - | - | - | - | - | - | - |
| 2dpi vs. 21dpi | *p=*0.9969 | *p*>0.9999 | *p=*0.7674 | *p=*0.8106 | - | - | - | - | - | - | - |
| 3dpi vs. 4dpi | *p=*0.8633 | *p=*0.6119 | *p*>0.9999 | *p=*0.9101 | *p=*0.9327 | *p=*0.9116 | *p=*0.6783 | *p=*0.4441 | *p*>0.9999 | *p=*0.7145 | *p=*0.8707 |
| 3dpi vs. 5dpi | *p=*0.8841 | *p=*0.8956 | *p=*0.2551 | *p=*0.9007 | *p=*0.1751 | *p=*0.8590 | *p*>0.9999 | *p*>0.9999 | *p*>0.9999 | *p*=0.9994 | *p*>0.9999 |
| 3dpi vs. 6dpi | *p=*0.9132 | *p=*0.7771 | *p=*0.9417 | *p=*0.9939 | *p=*0.9885 | *p=*0.9844 | *p=*0.9485 | *p*=0.6252 | *p*=0.9309 | *p*=0.8776 | *p*=0.9139 |
| 3dpi vs. 7dpi | *p=*0.7769 | *p=*0.9329 | *p=*0.7626 | *p=*0.9718 | *p=*0.9427 | *p=*0.9584 | *p=*0.9993 | *p*=0.9925 | *p*=0.8860 | *p*>0.9999 | *p*=0.9930 |
| 3dpi vs. 9dpi | *p=*0.5685 | *p=*0.4701 | *p=*0.9864 | *p=*0.9454 | *p=*0.9287 | *p=*0.9720 | *p=*0.8539 | *p*=0.6224 | *p*=0.9998 | *p*=0.8278 | *p*=0.8673 |
| 3dpi vs. 11dpi | *p=*0.6512 | *p=*0.8111 | *p*>0.9999 | *p=*0.6927 | *p=*0.7209 | *p=*0.6526 | *p=*0.6400 | *p*=0.6239 | *p*=0.4532 | *p*=0.6959 | *p*=0.8171 |
| 3dpi vs. 15dpi | *p=*0.5160 | *p=*0.9120 | *p=*0.8244 | *p=*0.6409 | *p=*0.6042 | *p=*0.5722 | *p=*0.8537 | *p*>0.9999 | *p*=0.2288 | *p*=0.7636 | *p*=0.6933 |
| 3dpi vs. 21dpi | *p=*0.7558 | *p=*0.8867 | *p=*0.8585 | *p=*0.9029 | *p=0*.8616 | *p=*0.8254 | *p=*0.9571 | *p*=0.9477 | *p*=0.4332 | *p*>0.9999 | *p*=0.9952 |
| 4dpi vs. 5dpi | *p=*0.9352 | *p=*0.5102 | *p=*0.9005 | *p=*0.9192 | *p=*0.0727 | *p=*0.7489 | *p=*0.6089 | *p*=0.1717 | *p*=0.9861 | *p*=0.8840 | *p*=0.6011 |
| 4dpi vs. 6dpi | *p=*0.3792 | *p=*0.2814 | *p=*0.5377 | *p=*0.7203 | *p=*0.7988 | *p=*0.9991 | *p=*0.2506 | *p*=0.7666 | *p*=0.6429 | *p*=0.2203 | *p*=0.5377 |
| 5dpi vs. 6dpi | *p*>0.9999 | *p*>0.9999 | *p*=0.9993 | *p*=0.2345 | **p*=0.0288 | *p*=0.1363 | *p*=0.8922 | **p*=0.0215 | *p*=0.8840 | *p*=0.9874 | *p*=0.7988 |
| 7dpi vs. 9dpi | *******p*<0.0001** | *******p*<0.0001** | *******p*<0.0001** | *******p*<0.0001** | - | - | *******p*<0.0001** | *******p*<0.0001** | - | *******p*<0.0001** | *******p*<0.0001** |
| 7dpi vs. 11dpi | *******p*<0.0001** | *******p*<0.0001** | *******p*<0.0001** | *******p*<0.0001** | *******p*<0.0001** | *******p*<0.0001** | - | - | *******p*<0.0001** | - | - |
| 7dpi vs. 15dpi | - | - | - | - | *******p*<0.0001** | *******p*<0.0001** | - | - | *******p*<0.0001** | - | - |
| 7dpi vs. 21dpi | - | - | - | - | *******p*<0.0001** | *******p*<0.0001** | - | - | *******p*<0.0001** | - | - |
| 9dpi vs. 11dpi | *******p*<0.0001** | *******p*<0.0001** | *******p*<0.0001** | *******p*<0.0001** | - | - | - | - | - | - | - |
| 11dpi vs. 15dpi | - | - | - | - | *******p*<0.0001** | *******p*<0.0001** | *******p*<0.0001** | *******p*<0.0001** | *******p*<0.0001** | *******p*<0.0001** | *******p*<0.0001** |
| 11dpi vs. 21dpi | - | - | - | - | *******p*<0.0001** | *******p*<0.0001** | *******p*<0.0001** | *******p*<0.0001** | *******p*<0.0001** | *******p*<0.0001** | *******p*<0.0001** |
| 15dpi vs. 21dpi | *******p*<0.0001** | *******p*<0.0001** | *******p*<0.0001** | *******p*<0.0001** | *******p*<0.0001** | *******p*<0.0001** | *******p*<0.0001** | *******p*<0.0001** | *******p*<0.0001** | *******p*<0.0001** | *******p*<0.0001** |

^£^Tukey's multiple comparisons test was performed to compare means of a given antiviral cytokine and transcription factor gene expression at two timepoints in a given tissue (Figure 4 – 6). Significant statistical difference thresholds are **p* ≤ 0.05, ***p* ≤ 0.01, ****p* ≤ 0.001, *****p* ≤ 0.0001, ns = not significant (unmarked).
