# Supplementary material for: Tissue tropism, pathology, and pathogenesis of West Nile virus infection in saltwater crocodile (Crocodylus porosus)
Source: PLoS Negl Trop Dis. 2025 Aug 4;19(8):e0013385. doi: 10.1371/journal.pntd.0013385 (PMC12331170; doi:10.1371/journal.pntd.0013385)
Supplement: S13 Table — (DOCX) [file pntd.0013385.s013.docx]

**S13 Table.** Summary of p-values from Tukey's multiple comparisons test of the level of upregulated kidney cytokine and transcription factor expression between the different timepoints^£^

| **Timepoint** | **Antiviral genes** | | | | **Cell proliferation and apoptosis gene** | | **Pro and anti-inflammatory genes** | | | | |
| --- | --- | --- | --- | --- | --- | --- | --- | --- | --- | --- | --- |
|  | **IRF3** | **OASL** | **Mx1-like** | **TFEB** | **Ki67** | **CASP9** | **IL-1 β like** | **IL-34** | **HIF-1-α** | **CSF1[M-CSF]** | **TGF Beta** |
| 1dpi vs. 2dpi | *p=*0.9971 | *p=*0.9997 | >0.9999 | >0.9999 | *p=*0.9844 | *p=*0.8654 | *p*>0.9999 | *p*>0.9999 | *p*>0.9999 | *p=*0.9897 | *p=*0.9934 |
| 1dpi vs. 3dpi | *p=*0.8094 | *p=*0.7136 | *p=*0.9995 | *p=*0.9350 | *p=*0.9868 | *p=*0.4760 | *p=*0.6305 | *p=*0.6601 | *p=*0.8612 | *p=*0.3865 | *p=*0.9986 |
| 1dpi vs. 4dpi | *p=*0.9943 | *p=*0.9184 | *p=*0.9891 | *p=*0.6794 | *p=*0.7500 | *p=*0.9997 | *p=*0.9919 | *p=*0.9914 | *p=*0.9959 | *p*>0.9999 | *p=*0.6861 |
| 1dpi vs. 5dpi | *p=*0.2599 | *p=*0.4256 | *p=*0.9859 | *p=*0.7259 | *p=*0.6819 | *p=*0.2338 | *p=*0.3371 | *p=*0.2322 | *p=*0.9848 | *p=*0.2181 | *p=*0.9993 |
| 1dpi vs. 6dpi | *p=*0.9451 | *p=*0.9980 | *p=*0.6756 | *p=*0.7038 | *p=*0.8346 | *p*>0.9999 | *p=*0.9861 | *p=*0.9607 | *p=*0.2337 | *p*>0.9999 | *p=*0.6358 |
| 1dpi vs. 7dpi | - | - | - | - | *p=*0.0973 | *p=*0.0768 | - | - | *p=*0.0668 | - | - |
| 1dpi vs. 9dpi | *p=*0.1408 | ****p=*0.0211** | *p=*0.8538 | ****p=*0.0211** | *p=*0.0863 | *p=*0.0658 | *p=*0.0574 | *p=*0.0998 | ****p=*0.0308** | ****p=*0.0211** | *p=*0.1664 |
| 1dpi vs. 11dpi | *p=*0.7592 | ****p=*0.0227** | *p=*0.9983 | >0.9999 | *p=*0.9566 | *p=*0.2114 | - | - | *p=*0.9962 | - | - |
| 1dpi vs. 15dpi | *p=*0.2284 | ****p=*0.0211** | *p=*0.9993 | ****p=*0.0353** | - | - | - | - | - | - | - |
| 2dpi vs. 3dpi | *p=*0.9307 | *p=*0.6695 | *p=*0.9931 | *p=*0.8045 | *p=*0.7775 | *p=*0.8209 | *p=*0.6966 | *p=*0.7573 | *p=*0.8054 | *p=*0.5778 | *p=*0.6864 |
| 2dpi vs. 4dpi | *p*>0.9999 | *p*>0.9999 | *p=*0.8850 | *p=*0.8561 | *p=*0.9451 | *p=*0.9825 | *p=*0.9751 | *p=*0.9701 | *p=*0.9492 | *p=*0.5841 | *p=*0.8971 |
| 2dpi vs. 5dpi | *p=*0.9292 | *p=*0.6101 | *p=*0.8734 | *p=*0.7920 | *p=*0.7503 | *p=*0.7346 | *p=*0.7255 | *p=*0.7116 | *p=*0.9381 | *p=*0.6108 | *p=*0.8423 |
| 2dpi vs. 6dpi | *p*>0.9999 | *p*>0.9999 | *p=*0.5155 | *p=*0.8529 | *p=*0.9830 | *p=*0.9922 | *p=*0.9601 | *p=*0.9153 | *p=*0.1985 | *p=*0.8477 | *p=*0.8820 |
| 2dpi vs. 7dpi | - | - | - | - | *p=*0.2104 | *p=*0.2479 | - | - | *p=*0.0572 | - | - |
| 2dpi vs. 9dpi | *p=*0.3705 | *p=*0.2338 | *p=*0.8376 | *p=*0.1149 | *p=*0.1795 | *p=*0.2200 | *p=*0.1529 | *p=*0.2070 | ****p=*0.0260** | *p=*0.2199 | *p=*0.1271 |
| 2dpi vs. 11dpi | *p=*0.8989 | *p=*0.7989 | *p=*0.9860 | *p*>0.9999 | *p=*0.8787 | *p=*0.7938 | - | - | *p=*0.9965 | - | - |
| 2dpi vs. 15dpi | *p=*0.5250 | *p=*0.3399 | *p=*0.9998 | *p=*0.2406 | - | - | - | - | - | - | - |
| 3dpi vs. 4dpi | *p=*0.9709 | *p=*0.9001 | *p=*0.9211 | *p=*0.8631 | *p=*0.9100 | *p=*0.9406 | *p=*0.8991 | *p=*0.9187 | *p=*0.9542 | *p=*0.9195 | *p=*0.8275 |
| 3dpi vs. 5dpi | *p*>0.9999 | *p=*0.9996 | *p=*0.9178 | *p*>0.9999 | *p*>0.9999 | *p>*0.9999 | *p*>0.9999 | *p*>0.9999 | *p=*0.9600 | *p*>0.9999 | *p*>0.9999 |
| 3dpi vs. 6dpi | *p=*0.9398 | *p=*0.9495 | *p=*0.5238 | *p=*0.8604 | *p=*0.9327 | *p=*0.9541 | *p=*0.8932 | *p=*0.8899 | *p*>0.9999 | *p=*0.8700 | *p=*0.8254 |
| 3dpi vs. 7dpi | *p=*0.8316 | *p=*0.6149 | *p=*0.1617 | *p=*0.3305 | *p=*0.4930 | *p=*0.5778 | *p=*0.5009 | *p=*0.6413 | *p=*0.4806 | *p=*0.6369 | *p=*0.3209 |
| 3dpi vs. 9dpi | *p=*0.6891 | *p=*0.4687 | *p=*0.0577 | *p=*0.2027 | *p=*0.4112 | *p=*0.5057 | *p=*0.3755 | *p=*0.4868 | *p=*0.3186 | *p=*0.4820 | *p=*0.2380 |
| 3dpi vs. 11dpi | *p=*0.9969 | *p*>0.9999 | *p=*0.4873 | *p=*0.9810 | *p*>0.9999 | *p*>0.9999 | *p=*0.9999 | *p*>0.9999 | *p=*0.8114 | *p*>0.9999 | *p=*0.9904 |
| 3dpi vs. 15dpi | *p=*0.9673 | *p=*0.7403 | *p=*0.1308 | *p=*0.4560 | *p=*0.7466 | *p=*0.8117 | *p=*0.7110 | *p=*0.8072 | *p=*0.5019 | *p=*0.8062 | *p=*0.5416 |
| 3dpi vs. 21dpi | *p=*0.9708 | *p=*0.9923 | *p=*0.9761 | *p=*0.9484 | *p=*0.9992 | *p=*0.9999 | *p=*0.9912 | *p=*0.9984 | *p=*0.8859 | *p=*0.9948 | *p=*0.9355 |
| 4dpi vs. 5dpi | *p=*0.8612 | *p=*0.6237 | *p=*0.9525 | *p=*0.7387 | *p=*0.7313 | *p=*0.7029 | *p=*0.6803 | *p=*0.7150 | *p=*0.9525 | *p=*0.7730 | *p=*0.7431 |
| 4dpi vs. 6dpi | *p=*0.6772 | *p*>0.9999 | *p=*0.8400 | *p=*0.9525 | *p=*0.9525 | *p*>0.9999 | *p=*0.5377 | *p=*0.9814 | *p=*0.6146 | *p=*0.2345 | *p=*0.9525 |
| 5dpi vs. 6dpi | *p=*0.7552 | *p=*0.8003 | *p=*0.9525 | *p=*0.7496 | *p=*0.7860 | *p=*0.7739 | *p=*0.6772 | *p=*0.6364 | *p=*0.9422 | *p=*0.6993 | *p=*0.7531 |
| 7dpi vs. 9dpi | - | - | - | - | *******p*<0.0001** | *******p*<0.0001** | - | - | *******p*<0.0001** | - | - |
| 7dpi vs. 11dpi | - | - | - | - | *******p*<0.0001** | *******p*<0.0001** | *******p*<0.0001** | *******p*<0.0001** | *******p*<0.0001** | *******p*<0.0001** | *******p*<0.0001** |
| 7dpi vs. 15dpi | - | - | - | - | - | - | *******p*<0.0001** | *******p*<0.0001** | - | *******p*<0.0001** | *******p*<0.0001** |
| 7dpi vs. 21dpi | *******p*<0.0001** | *******p*<0.0001** | *******p*<0.0001** | *******p*<0.0001** | - | - | *******p*<0.0001** | *******p*<0.0001** | - | *******p*<0.0001** | *******p*<0.0001** |
| 9dpi vs. 11dpi | *******p*<0.0001** | *******p*<0.0001** | *******p*<0.0001** | *******p*<0.0001** | *******p*<0.0001** | *******p*<0.0001** | - | - | *******p*<0.0001** | - | - |
| 9dpi vs. 15dpi | *******p*<0.0001** | *******p*<0.0001** | *******p*<0.0001** | *******p*<0.0001** | - | - | - | - | - | - | - |
| 11dpi vs. 15dpi | *******p*<0.0001** | *******p*<0.0001** | *******p*<0.0001** | *******p*<0.0001** | - | - | *******p*<0.0001** | *******p*<0.0001** | - | *******p*<0.0001** | *******p*<0.0001** |
| 11dpi vs. 21dpi | - | - | - | - | - | - | *******p*<0.0001** | *******p*<0.0001** | - | *******p*<0.0001** | *******p*<0.0001** |
| 15dpi vs. 21dpi | - | - | - | - | *******p*<0.0001** | *******p*<0.0001** | *******p*<0.0001** | *******p*<0.0001** | *******p*<0.0001** | *******p*<0.0001** | *******p*<0.0001** |

^£^Tukey's multiple comparisons test was performed to compare median of a given antiviral cytokine and transcription factor gene expression at two timepoints in a given tissue (Sup. Figure 4 – 6). Significant statistical difference thresholds are **p* ≤ 0.05, ***p* ≤ 0.01, ****p* ≤ 0.001, *****p* ≤ 0.0001, ns = not significant (unmarked).
