# Supplementary figures and images for: Tissue tropism, pathology, and pathogenesis of West Nile virus infection in saltwater crocodile (Crocodylus porosus)
Source: PLoS Negl Trop Dis. 2025 Aug 4;19(8):e0013385. doi: 10.1371/journal.pntd.0013385 (PMC12331170; doi:10.1371/journal.pntd.0013385)

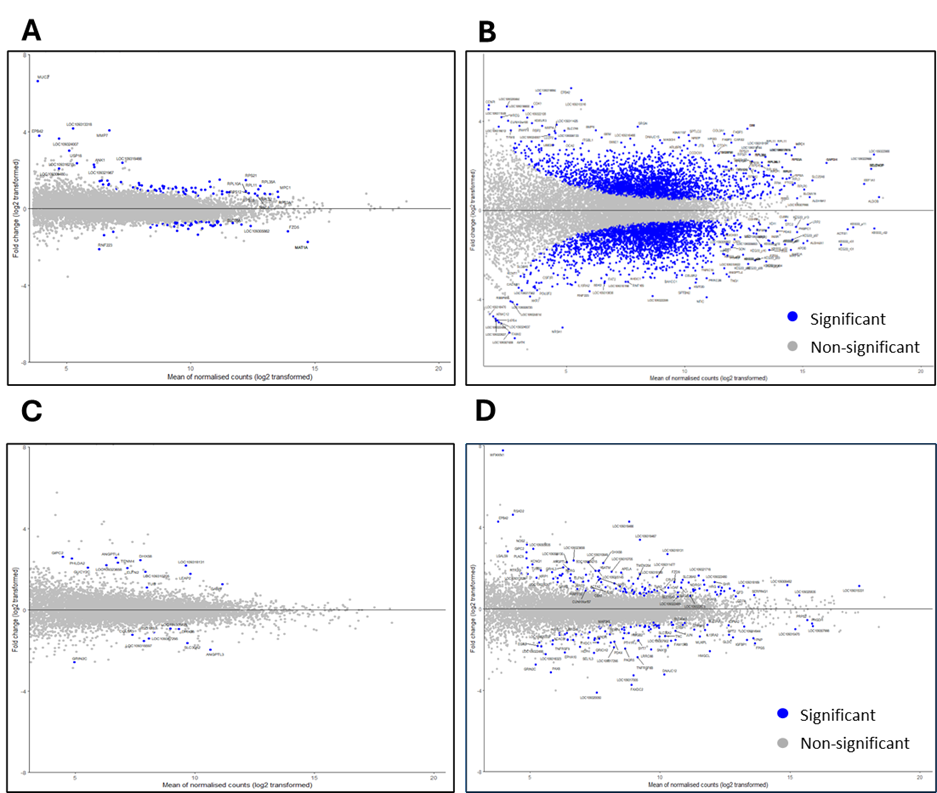

Supplement: S1 Fig — (A and B) MA-plot for early and late response to infection in kidney. (C and D). MA-plot for early and late response to infection in liver. (TIF) [file pntd.0013385.s014.tif]

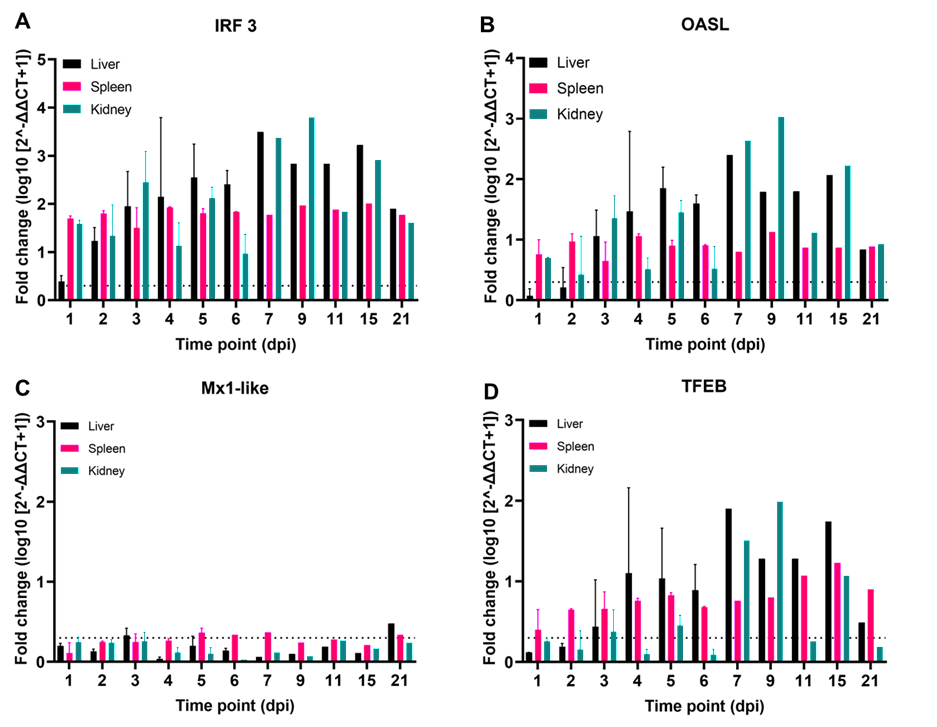

Supplement: S2 Fig — The discontinuous line represents baseline transcription (one-fold-change equivalent to cytokine gene expression in mock-infected animal). The statistical comparison of the transcriptional profile of individual cytokine and transcription factor genes at different time points in various tissues was tested by a two-way ANOVA carried out for multiple comparison analysis with the α-level set at 0.05 with a Tukey’s post-test, with individual variances computed for each comparison. To compare the overall transcriptional profile of cytokine and transcription factor genes in tissues, ordinary two-way ANOVA with a Tukey’s multiple comparisons test, with a single pooled variance. (TIF) [file pntd.0013385.s015.tif]

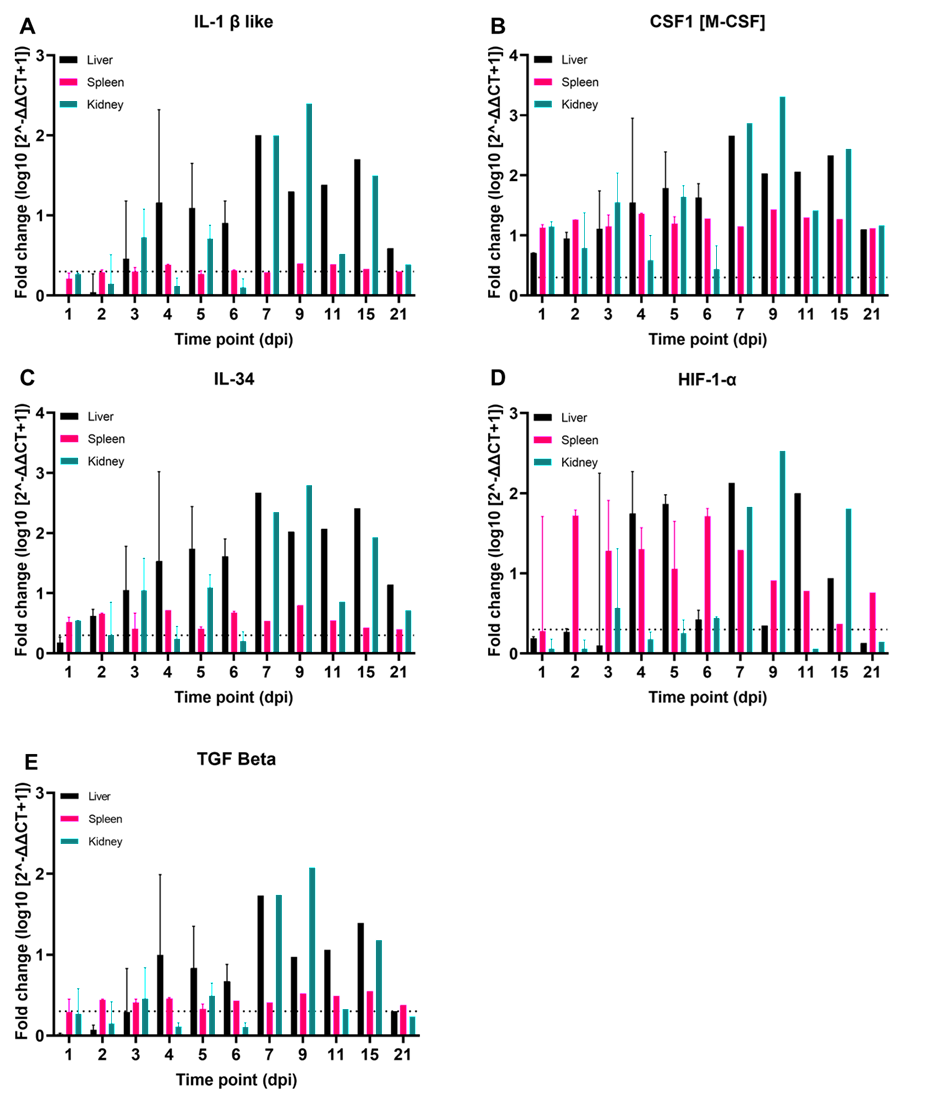

Supplement: S3 Fig — The discontinuous line represents baseline transcription (one-fold change equivalent to cytokine gene expression in mock infected animal). The statistical comparison of the transcriptional profile of individual cytokine genes at different time points in various tissues was tested by a two-way ANOVA carried out for multiple comparison analysis with the α-level set at 0.05 with a Tukey’s post-test, with individual variances computed for each comparison. To compare the overall transcriptional profile of cytokine genes in tissues, ordinary two-way ANOVA with a Tukey’s multiple comparisons test, with a single pooled variance. (TIF) [file pntd.0013385.s016.tif]

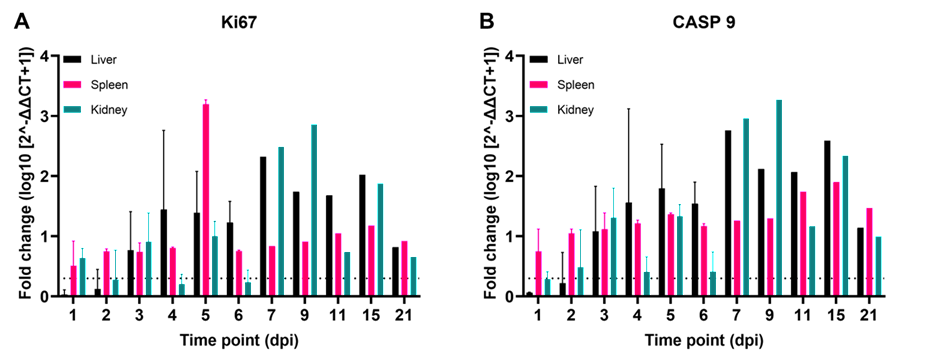

Supplement: S4 Fig — The discontinuous line represents baseline transcription (one-fold change equivalent to cytokine gene expression in mock-infected animal). The statistical comparison of the transcriptional profile of individual cytokine genes at different time points in various tissues was tested by a two-way ANOVA carried out for multiple comparison analysis with the α-level set at 0.05 with a Tukey’s post-test, with individual variances computed for each comparison. To compare the overall transcriptional profile of cytokine genes in tissues, ordinary two-way ANOVA with a Tukey’s multiple comparisons test, with a single pooled variance. (TIF) [file pntd.0013385.s017.tif]

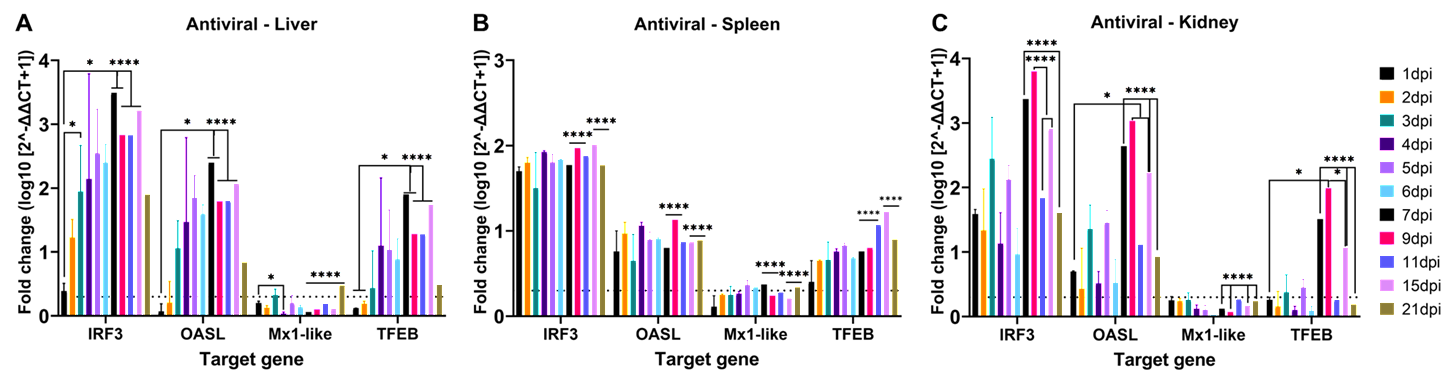

Supplement: S5 Fig — A discontinuous line at log10 (Y + 1) represents baseline transcriptional profile (one-fold change equivalent to cytokine and transcription factor gene expression in mock-infected animals). The Tukey’s multiple comparisons test was performed to compare median of antiviral cytokine and transcription factor gene expression at various time points in each tissue. Significant statistical difference thresholds are *p ≤ 0.05, **p ≤ 0.01, ***p ≤ 0.001, ****p ≤ 0.0001, ns = not significant. (TIF) [file pntd.0013385.s018.tif]

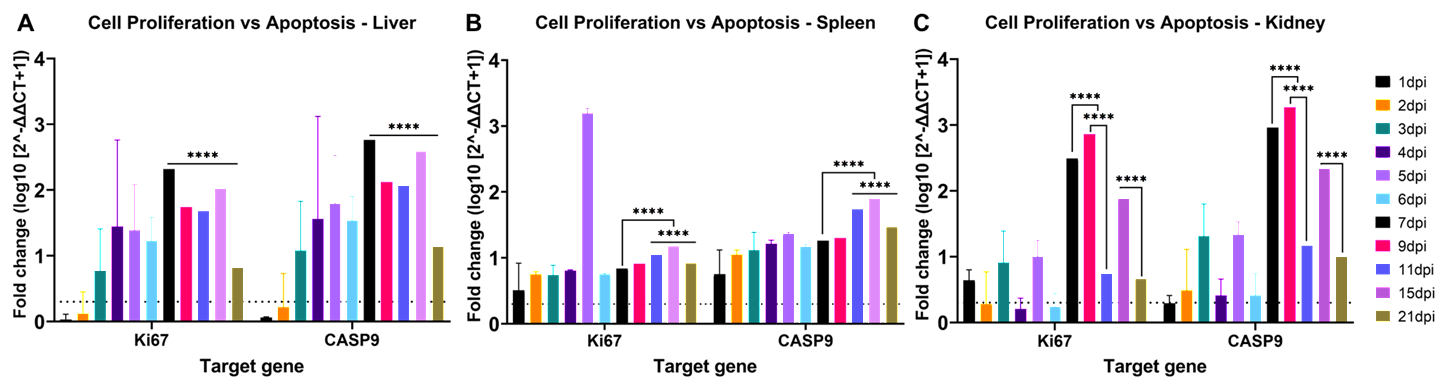

Supplement: S6 Fig — A discontinuous line at log10 (Y + 1) represents baseline transcriptional profile (one-fold change equivalent to cytokine gene expression in mock-infected animal). The Tukey’s multiple comparisons test was performed to compare median of cell proliferation and apoptosis cytokine gene expression at various time points in each tissue. Significant statistical difference thresholds are *p ≤ 0.05, **p ≤ 0.01, ***p ≤ 0.001, ****p ≤ 0.0001, ns = not significant. (TIF) [file pntd.0013385.s019.tif]

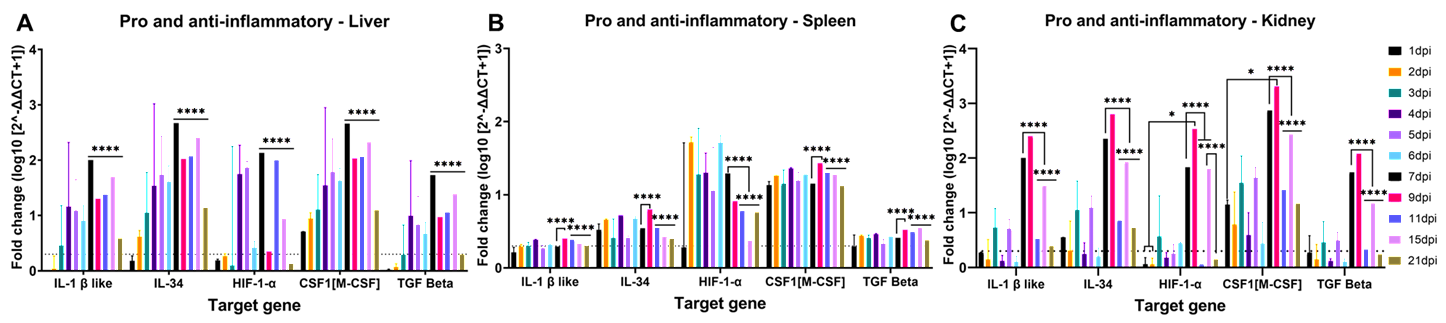

Supplement: S7 Fig — A discontinuous line at log10 (Y + 1) represents baseline transcriptional profile (one-fold change equivalent to cytokine gene expression in mock infected animals). The Tukey’s multiple comparisons test was performed to compare median of cell proliferation and apoptosis cytokine gene expression at various time points in each tissue. Significant statistical difference thresholds are *p ≤ 0.05, **p ≤ 0.01, ***p ≤ 0.001, ****p ≤ 0.0001, ns = not significant. (TIF) [file pntd.0013385.s020.tif]
